# Benthic bacteria communities of coral reefs are shaped by sediment properties rather than coral trophic state

**DOI:** 10.1371/journal.pone.0346135

**Published:** 2026-04-03

**Authors:** Ana Olmos Pin, Graeme F. Clark, Raphael Burkart-Radtke, Jakob Quade, Stephanie G. Gardner

**Affiliations:** 1 School of Life and Environmental Sciences, The University of Sydney, Camperdown, New South Wales, Australia; 2 The University of Ghent, Ghent, Belgium; King Abdulaziz University, SAUDI ARABIA

## Abstract

Understanding how different reef components contribute to ecosystem stability and function is crucial for assessing the health of reefs. Reef sediments and their associated bacteria are essential for reef productivity and stability, but are often overlooked in reef assessments. Here we examined bacterial communities and environmental properties in sediments closely associated with the coral *Porites lutea*, compared to those in adjacent sediment controls at One Tree Island Reef, and used incubation chambers to assess metabolic rates and nutrient availability. We found that seawater enclosed in the chamber containing *P. lutea* coral-associated sediment exhibited significantly higher gross photosynthetic and respiration rates than seawater enclosed in the chamber as sediment only controls, and the coral-associated bacterial assemblages within the sediment had higher alpha diversity and richness metrics. Additionally, bacterial communities were primarily shaped by total nitrogen, which was the strongest predictor of microbial community. Differential abundance analyses identified the families Woeseiaceae, Pirellulaceae, and Streptomycetaceae increased with higher photosynthetic and respiratory activity, whereas Rhodobacteraceae and Alteromonadaceae showed contrasting responses to carbon and nitrogen content. Together, these results suggest reef sediment bacterial assemblages were primarily structured by sediment properties and benthic productivity, rather than coral trophic state. This highlights the role of sediment physicochemical gradients in shaping and maintaining microbial diversity and ecosystem function.

## Introduction

To effectively protect coral reef ecosystems, it is crucial to understand how each component of the reef contributes to the ecological stability and function of the ecosystem [1]. Reef health assessments primarily focus on monitoring the status of reef-building corals, algae cover, and the diversity of associated benthic and pelagic fauna, including sponges, invertebrates, and fish [2,3], but often overlook reef sediments, which cover approximately 95% of Australia’s Great Barrier Reef [4].

Reef sediments derive mainly from the erosion and bioerosion of living and dead corals by specific fish species, particularly parrotfish [5,6], as well as direct inputs by carbonate-secreting organisms, such as foraminifera, molluscs and calcareous algae [7–9]. Thus, most of the sediment is produced *in situ* and is primarily composed of calcium carbonate, particularly in lagoonal reef sediment [10,11]. Lagoon floors comprise 61% of the Great Barrier Reef and are inhabited by highly diverse macro- and microorganisms [4]. Studies on the Great Barrier Reef suggest that the sediment microbiome is a useful indicator of environmental perturbations in coral reef ecosystems [12–14].

The sediment microbiome comprises eukaryotic and prokaryotic organisms, with bacteria being the most abundant among the prokaryotes [15]. Their growth is enhanced by the porous nature of carbonate sands, offering a higher specific surface area than silicate sands [16,17]. As such, sediment in the lagoonal coral reef habitat promotes a diverse bacterial community in both composition and functionality [17,18], being key in biogeochemical cycles [19] and coral-sediment interactions [20–22]. Although sediment bacteria are considered vital for the overall functioning and balance of coral reef ecosystems [23,24], surprisingly little is known about the bacterial communities of benthic marine sediments and their potential influence on overall reef health [25].

Within the sediment, bacteria significantly contribute to the remineralisation of organic matter, like coral mucus, and the recycling of nutrients [26,27], reducing the loss of energy and nutrients from the ecosystem [28]. More specifically, calcareous sediment bacteria play an essential role in the nitrogen cycle [29,30]. Nitrogen-fixing bacteria (diazotrophs) in the sediment can significantly contribute to the nitrogen budget of the coral reef by adding bioavailable nitrogen to the system as ammonium, while nitrifying bacteria prevent nitrogen loss by producing *de novo* bioavailable nitrogen as nitrate [30]. Both pathways are essential for primary productivity in oligotrophic (nutrient-poor) environments, such as coral reefs [23,31,32], and occur in the upper few centimetres of reef sediments [33].

The critical role of microbes in corals is increasingly understood, as they facilitate host function, adaptability, and resilience to environmental stressors [34,35]. To date, research on the microbiome of coral reef ecosystems has focused on assessing microbial diversity and function related to corals [20,21,36,37], sponges [38,39], fish and seagrass [40], and the surrounding water [14], while knowledge of the sediment microbiome remains limited. Sediment bacteria may act as a reservoir of symbiotic bacteria for corals, potentially influencing corals’ health, function and capacity to adapt to environmental changes [20,41]. For example, Glasl *et al.* [22] found that the aged mucus-associated bacteria community overlapped with that of the new mucus and sediment bacterial communities, suggesting that the nutrient-rich coral mucus interacts with the upper sediment layers through resuspension. While sediment can harbour beneficial bacteria for corals, it can also be a direct source of pathogenic bacteria, including those from the Rhodobacterales and Rhizobiales families, linked to Stony Coral Tissue Loss Disease (SCTLD) lesions [42,43]. Characterising bacterial communities in coral-adjacent sediments is therefore essential for understanding the potential interactions between sediments and coral microbiomes.

Advances in next-generation sequencing, combined with the recognition of bacteria as key players in coral reef ecosystems, have led to an increased commitment to narrowing the knowledge gap on benthic marine sediment microbial dynamics [2]. In this study, the 16S ribosomal RNA (rRNA) gene was used to determine the bacterial community composition of sediments closely associated with *Porites lutea* corals (i.e., coral-associated sediment) and compare these to sediment only controls within a pristine reef lagoon at One Tree Island (OTI), southern Great Barrier Reef. *In situ* incubations of chambers with the coral-associated sediment and sediment controls were conducted to measure seawater respiration (R) and gross primary production (GPP) rates, assessing the trophic state of *P. lutea* as a proxy for overall coral health [44,45].

This study had three main objectives (i) to characterise the composition and diversity of sediment bacterial communities associated with healthy *P. lutea* coral colonies and compare this with sediment only controls, (ii) to identify key bacterial taxa that drive community compositional differences, and (iii) to determine the influence of sediment environmental parameters (e.g., nitrogen content, granulometry) and coral functional traits (e.g., GPP:R ratio, surface area, proximity to patch reefs) on bacterial community structure. Our study provides new insights into the factors shaping reef sediment microbiota and establishes a critical baseline for future monitoring of sediment microbial dynamics under a changing climate.

## Materials and methods

### Study site

**Fig 1:**
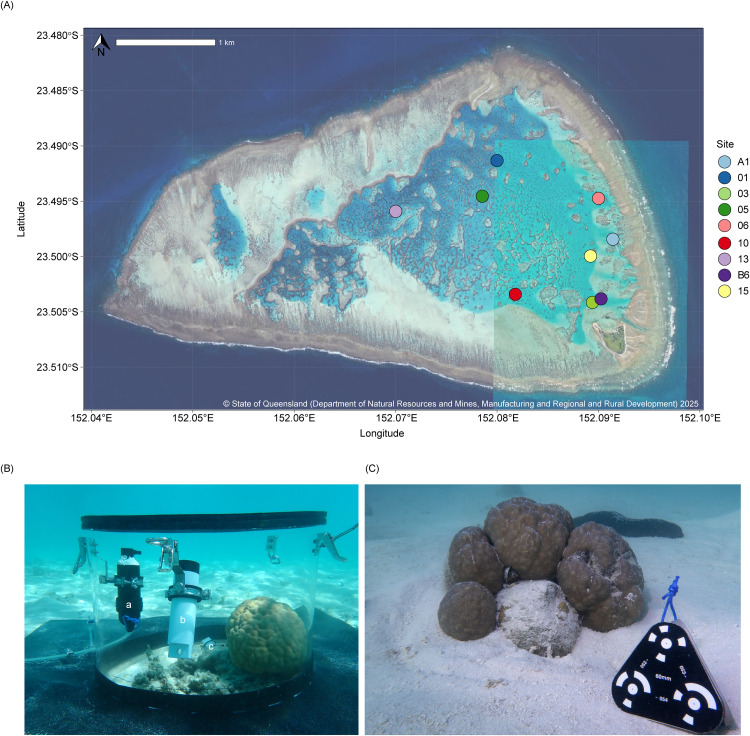
Locations of the *in situ* incubation sites and representative *Porites lutea* colony at One Tree Island Reef (Lagoon 1). **(A)** Aerial photography of OTI Reef with coloured symbols indicating the location of the *Porites lutea* coral colonies and the sediment only chambers, which were selected for *in situ* incubation within Lagoon 1. Source of OTI Reef map: Queensland Globe, © State of Queensland (Department of Natural Resources and Mines, Manufacturing and Regional and Rural Development) 2025, licensed under CC BY 4.0 licence. **(B)** The transparent *in situ* incubation chamber over a *Porites lutea* coral, showing the (a) water pump for continuous water circulation, (b) oxygen logger to measure seawater dissolved oxygen and (c) light and temperature logger. **(C)** Example of a *Porites lutea* coral colony used for coral incubations. Scale bars (black-and-white triangle objects) were placed near each coral for 3D reconstruction of the coral in the modelling software.

This study was carried out during the austral spring (Sep/Oct) of 2023 at OTI Reef, a lagoonal platform reef part of the Capricorn-Bunker Group near the southern boundary of the Great Barrier Reef (23°30′S 152°06′E), Australia (Fig 1A). OTI Reef is an irregularly shaped pseudo-atoll, 5.5 km long and up to 3.5 km wide. The reef crest encloses three shallow lagoons (2–10 m deep) and a coral cay in the southeastern corner of the reef crest. All research activities were conducted within Lagoon 1, one of the three enclosed lagoons.

### Benthic incubation chamber and sampling procedure

*In situ* incubation chambers were constructed as described by Roth *et al.* [45] and deployed over coral heads (Fig 1B). Briefly, each incubation chamber consisted of a clear, cylindrical polycarbonate chamber body (diameter 0.5 m, height 0.39 m), closed at one end by a removable gas-tight lid. Inside, a MiniDO_2_T oxygen Logger (Precision Measurement Engineering Inc., California, USA) and a HOBO^®^ Pendant light and temperature logger (UA-002; Onset Computer Corporation, Massachusetts, USA) were installed in each chamber to measure oxygen flux of the seawater contained within the chamber. Water circulation in the chamber was set to ~ 2 L/min by a custom-made submersible pump (6V DC motor, 300mA, 2W) using a magnetic impeller (powered by 4 x 1.5V AA batteries). All chambers had a natural rubber skirt (3 mm thick) fitted to the chamber body to isolate the chamber from the surrounding water and improve the seal to the sandy bottom.

Two SCUBA divers deployed the incubation chambers over twenty haphazardly selected *Porites lutea* coral heads over nine sites within the largest lagoon at OTI (Fig 1)*. P. lutea* is a dominant reef-building coral, widely distributed in the Indo-Pacific. The coral heads were growing on sand near patch reefs at a depth of 4–5 m, showed no visible signs of bleaching or disease, were suitably sized to fit inside the incubation chamber and a secure seal was possible around the chamber (i.e., no nearby rocks or reef structure that would prevent pushing the incubation chamber 5 cm deep into the sand to properly seal for the duration of the incubation period). First, the GPS location for each coral head was recorded, then chambers without lids were carefully positioned over the coral heads of interest and pushed into the sediment to a depth of approximately 5 cm. The silicone skirt was gently lowered until resting on top of the sediment. Chambers were left untouched for at least 1 hour to allow the suspended sediment to settle. Subsequently, the oxygen loggers and water circulation pumps were secured in brackets inside the open chambers, and temperature-light sensors were deployed on the sediment (Fig 1B). Each coral head underwent two incubation periods: one dark and one light, each lasting ~2 hours, with a one-hour interval between the two incubation periods. The dark incubation commenced once blackout material was placed over the sealed chamber, and the light incubation was initiated by sealing the chamber. The start and end times of both incubations were recorded for the posterior analysis of the community oxygen flux. Oxygen, temperature and light inside the chamber were recorded every minute.

After the dark incubation, the lid was removed, and the top 1 cm of sediment in the chamber was sampled into three 15 mL Falcon tubes for 16S rRNA gene amplicon sequencing, minimising disruption of the surface sediment layer. Each incubation chamber containing coral (n = 20) was sampled in triplicate, yielding a total of n = 60. Incubation chambers on bare sediment (i.e., sediment-only controls) were deployed as a control for the coral-associated chambers. Each sediment control incubation chamber (n = 3) was sampled in triplicate at all nine sites, for a total of n = 81. These were deployed 12 m from a patch reef to avoid potential effects from the reef. One sediment core (PVC sediment core, 5x5 cm, collected within a 50 cm radius of the incubation chamber) was sampled for grain size and nutrient analysis. All samples were stored on ice after collection, for transport back to the Research Station (within 1 hour of collection). Samples were collected under Great Barrier Reef Marine Park Authority permit G23-48125.1 (issued to SGG).

At the OTI research station, sediment samples for 16S were homogenised and sub-sampled into 1.5 mL Eppendorf tubes. RNAlater (Thermo Fisher Scientific Inc, Massachusetts, USA) was added to each replicate to preserve the DNA and samples were stored frozen at −20°C. Sediment cores were left to settle before seawater was decanted off the top and the remaining sample was transferred to a zip-lock bag and frozen at −20 °C. All remaining analyses (detailed below) were conducted at the University of Sydney.

### Photometric measurements

Using photogrammetry and computer modelling techniques described in Ferrari *et al.* [46], the three-dimensional surface area (SA_3D_) and volume (V_C_) of each coral head were calculated to estimate their contribution to photosynthesis (P) and respiration (R) rates. Corals were photographed *in situ* by SCUBA divers from multiple angles using an Olympus Tough TG-6 digital camera and a ring light for illumination. Scale bars were placed near each coral head to enable accurate scaling for 3D reconstruction (Fig 1C). The image processing software Adobe Lightroom (Adobe Inc., California, USA) was used to adjust the white balance, and the 3D model of coral heads was built using the photogrammetry software Agisoft Metashape (Agisoft LLC, St. Petersburg, Russia) using default settings. The SA_3D_ (m^2^) and the V_C_ (m^3^) of coral heads were calculated.

For the calculation of metabolic rates, the effective volume (V_E_; the volume of seawater) in each chamber during incubation was obtained by the formula:


$$V_{E}\;(L)=V_{T}-(V_{L}+V_{P}+V_{C})$$

The coral volume (V_C_; converted to litres), oxygen logger volume (V_L_; in litres) and water pump volume (V_P_; in litres) were subtracted from the total seawater volume (V_T_; in litres) enclosed by the chamber above the sediment.

### Gross primary production and respiration analysis

Photosynthesis and respiration rates were processed and analysed using the *respR* package [47] in computing environment R [v.4.2.1; 48]. The metabolic rates were obtained in an unbiased manner using the *auto_rate* function, which automatically identifies the most linear regression of seawater oxygen concentration over time within each separate two-hour incubation, based on kernel density estimation. GPP (gross oxygen production rate during the light period) and R (oxygen consumption rate during the dark period) were extracted as the regression coefficient (slope). The regression coefficient represents the linear increase or decrease of seawater dissolved oxygen (DO) concentration inside the incubation chambers during both incubation periods (light and dark, respectively).

The *convert_rate* function was used to convert the unitless rates to the area-specific output unit: $\frac{mg\;{of\;O}_{\text{2}}}{m^{\text{2}}*h}\;$. Briefly, the seawater community DO flux was calculated as follows:

$DO\;flux=\;\frac{\left(\Delta O_{\text{2}}*V_{E}\right)}{\left(A_{E}*\Delta t\right)}$,

where ΔO_2_ (mg L^-1^) is the difference in DO concentration between the first (t0) and last point (t1) of the automatically identified linear regression, V_E_ is the effective seawater volume enclosed in the chamber, Δt is the period between the first and last point of the automatically identified linear regression, and A_E_ (m^2^) is the active photosynthetic surface area within the incubation chamber. For the coral-associated chambers, A_E_ was calculated as the 3D coral surface area (SA_3D_) plus the sediment surface area enclosed within the chamber, minus the basal coral surface area (i.e., the area in contact with the sediment), and as sediment surface area within the chamber for the sediment control samples, to ensure only the surfaces actively contributing to photosynthesis were included in the normalisation of gross photosynthetic and respiration rates. SA_3D_ of the community enclosed by the chamber. V_E_ and the SA_3D_ of the coral head were used to normalise the rate values. Rate values of both GPP and R were calculated and converted to the final area-specific unit: $\frac{g\;{of\;O}_{\text{2}}}{m^{\text{2}}*h}$.

Additionally, net primary production (NPP = GPP – R) and the photosynthesis-to-respiration ratio (P:R) were calculated to determine the dominant metabolic pathway, assuming that the nighttime respiration rate equals the daytime respiration rate [45,49]. A P:R ratio greater than one indicates that autotrophic activity predominates over heterotrophic activity (P:R > 1), and conversely when the ratio is less than one.

Differences between gross photosynthetic rates and respiration rates were tested using non-parametric Kruskal-Wallis tests, after checking and confirming normality and homogeneity of variances with the Shapiro-Wilk test and Levene’s test, respectively. The effects of sample type (coral-associated sediment and sediment control) and site were each tested for the gross photosynthetic and respiration rates separately.

### Grain size analysis

Evaluation of the grain size distribution of the sediment is necessary to determine the availability of organic matter (OM) in the sediment. OM transport by pore-water advection is more efficient in poorly sorted calcareous sands, so its availability differs from that in permeable carbonate sand and mud sediments [50,51].

Prior to analysis, sediments were freeze-dried for 48 h (Alpha 1–4 LSCbasic; Christ, Osterode am Harz, Germany) at 1 bar and sieved to obtain the < 2 mm fraction. The total dry mass, as well as the < 2 mm and > 2 mm fractions were recorded, and the gravel content (% of grains > 2 mm) was calculated. The following sediment grain-size and nutrient analyses were conducted on the sediment fraction below 2 mm.

Sediment grain size distribution was determined by laser particle size analysis using a Malvern Mastersizer 3000 (Malvern Panalytical Ltd, Malvern, UK). Samples were sub-sampled and analysed in triplicate. Before analysis, each replicate was digested in hydrogen peroxide to remove OM (30% H_2_O_2_, 6 mL, 48 hours). Excess hydrogen peroxide was removed by washing the replicates twice with water. In this study, a refractive index of 1.6 and an absorbance index of 0.01 were used, as the sediment composition primarily consisted of calcium carbonate. Malvern output data were processed in GRADISTAT [v.9.1; 52] to acquire the logarithmic graphical mean (descriptive term), standard deviation (sorting), skewness and kurtosis, calculated according to the formulas of Folk and Ward [53] based on the φ scale [54]. Mean values were calculated from the three technical replicates to determine the grain size characteristics of each sample.

### Sediment nutrient measurements

Sieved sediments were ground using a porcelain mortar and pestle and weighed into aluminium capsules. Samples were combusted in a macro elemental analyser (vario MACRO cube; Elementar Analysensysteme GmbH, Langenselbold, Germany), and total carbon (TC) and total nitrogen (TN) were measured as the amount of CO_2_ and nitrogen released by gas chromatography. Final values are expressed as the percentage of TC or TN in dry sediment since this method does not differentiate between organic and inorganic forms of the element. In most sediments, inorganic nitrogen is a minimal fraction of TN, so it can be assumed that organic nitrogen equals TN [19]. Meanwhile, the inorganic carbon fraction in calcareous sediments can constitute a significant proportion of the total carbon in the form of calcium carbonate. Therefore, sediment organic matter was estimated by loss on ignition [55]; 250 mg of sediment was subsampled into a glass tube and heated to 375 ºC in a furnace for 24 h [56]. Lastly, the total organic carbon to total nitrogen (C:N) ratio was calculated to assess the impact of sediment biogeochemical processes.

### DNA extraction, polymerase chain reaction (PCR) and amplicon sequencing

Total DNA was isolated from 0.35 g of homogenised sediment using the DNeasy PowerSoil Kit (QIAGEN, Hilden, Germany) following the manufacturer’s protocol and DNase-free water was used to elute the DNA. Genomic DNA was extracted from 60 sediment samples (from 20 individual incubations on coral heads, each with 3 replicates per incubation chamber), and 81 sediment control samples, as well as three blanks (extraction kit controls). Extracted DNA was stored at −20 °C before subsequent analyses.

Metabarcoding was performed on the hypervariable region V3–V4 of the 16S rRNA gene, which was chosen as a marker for the phylogenetic classifications of the microbial community. Amplicon libraries were created using the primer pair 341F (341 F: 5′-TCGTCGGCAGCGTCAGATGTGTATAAGAGACAG-CCTACGGGNGGCWGCAG-3′) and 785R (785 R: 5′-GTCTCGTGGGCTCGGAGATGTGTATAAGAGACAG- GACTACHVGGGTATCTAATCC-3′) primers with Illumina overhang adapter sequences [57,58].

The DNA samples were amplified in a single-step, 35-cycle PCR using a Mastercycler^®^ nexus X2 gradient PCR thermal cycler (Eppendorf South Pacific Pty. Ltd., Macquarie Park, Australia) under the following conditions: (i) initial denaturation at 94 °C for 2 min, followed by (ii) 35 cycles of denaturation at 94 °C for 30 s, annealing at 55 °C for 30 s and extension at 72 °C for 40 s, with (iii) a final elongation at 72 °C for 7 min. Each PCR reaction consisted of a total final volume of 25 µL, containing 12.5 µL of EconoTaq PLUS GREEN 2X Master Mix (Astral Scientific Pty. Ltd., Tarren Point, Australia), 0.75 µL of each forward and reverse primer, 8.5 µL of DNase-free water, and 2.5 µL of extracted DNA. Indexed and pooled PCR amplicons were sequenced on the 2 x 300 bp Illumina MiSeq sequencing platform at The Ramaciotti Centre for Genomics (The University of New South Wales, Australia). Extraction kit blanks (n = 3) and PCR negative controls (n = 3) were also sequenced.

### Statistical analysis of amplicon sequencing data

Adapter and primer sequences were removed using Cutadapt [v.4.4; 59] executed under Python [v.3.9.2; 60]. To ensure that only high-quality, primer-free reads and resulting amplicon sequences were analysed, data were processed in R [v.4.2.1; 48] following the bioinformatic pipeline *DADA2* workflow [61]. Briefly, paired-end reads were de-noised, and chimeras were removed using DADA2 to produce amplicon sequence variants (ASVs). ASVs were taxonomically classified using a Bayesian Last Common Ancestor algorithm [BLCA; 62] against the Genome Taxonomy Database R214 (GTDB [63]; released 9 June 2023: https://gtdb.ecogenomic.org/). ASV sequences were aligned with MAFFT [64], and a phylogenetic tree was generated using FastTree2 [65] on the CIPRES v.3.3 Science Gateway [66].

Prior to the data analyses, ASV contaminants were identified using the prevalence method in the *decontam* package [v.1.6.0; 67]. A total of 213 ASVs were identified as potential contaminants, detected in sequenced extraction blanks and PCR-negative controls, and were removed prior to downstream analyses. ASVs were analysed with *Vegan* package [v.2.6.4; 68] and *microeco* package [v.1.15.0; 69] in R.

### Coral-associated and sediment control sample comparison

These analyses were conducted on the full dataset (i.e., the sediment from inside incubation chambers with corals, n = 60, and the sediment from empty chambers, n = 81) to compare the coral-associated with sediment only control (non-coral-associated) sediments. Principal coordinates analysis (PCoA) was used to visualise dissimilarities in microbial communities between coral-associated and sediment-only samples, using weighted UniFrac distances that integrate the relative abundance of each ASV and phylogenetic distance [70]. Differences between bacterial communities of the coral-associated and sediment control samples were tested using PERMANOVA on weighted UniFrac distances with the *adonis2* function in Vegan. To account for unbalanced sampling and site-level structuring, marginal (Type III) sums of squares were used (by = “margin”) in a model including sample type (i.e., coral-associated and sediment control) and site (unifrac ~ sampletype + site). A second analysis constrained permutations within sites to test whether coral-associated and sediment control samples differed consistently within sites. Homogeneity of multivariate dispersion was evaluated using *betadisper* to assess whether differences in sample type (i.e., coral-associated and sediment control) were influenced by variation in dispersion.

To compare alpha diversity indices for the coral-associated and sediment-only samples, samples were rarefied to the smallest sample (1,134 reads). Homogeneity of variance was tested using Levene’s test, and normality assessed using Shapiro-Wilk test, before running a non-parametric Kruskal-Wallis test for all indices (i.e., observed number of ASVs, Shannon, Simpson and Chao1). The composition of the sediment bacteria community in each sample for the coral-associated and sediment only samples was shown for the top ten families (relative abundance).

To test for differentially abundant taxa between the coral-associated and sediment control samples, we used *DESEq2* package [v.1.38.3; 74], which uses negative binomial generalised linear model (GLM). Analysis was conducted at the family level taxonomic aggregation and the top ten with the largest positive and negative log_2_ Fold Changes (log_2_ FC) were plotted to illustrate the most representative bacterial families.

Core bacterial families, i.e., including only taxa found in 100% of the samples at the family level, were identified for the coral-associated versus the sediment-only samples. The core community was summarised as the mean relative abundance at the family level and visualised using a stacked bar plot. Core microbiome patterns were plotted using the *ggvenn* package [v.0.1.10; 71] to show the shared and unique core bacterial taxa between sample groups.

### Coral-associated sediment sample analyses

For the remaining analyses using the coral-associated samples (n = 60, with the 81 sediment-only samples removed), principal coordinates analysis (PCoA) was used to visualise dissimilarities in microbial communities using weighted UniFrac distances. Differences in bacterial community composition among coral-associated samples were assessed using permutational multivariate analysis of variance (PERMANOVA) based on weighted UniFrac distances with the *adonis2* function (Vegan), using 9,999 permutations. To account for the unbalanced, hierarchical design of the chambers nested within sites, marginal (Type III) sum of squares (by = “margin”) were calculated in the model (adonis2(unifrac ~ SA + Site/Chamber-replicate, data = sampledf, permutations = 9,999, by = “margin”). This allowed independent assessment of the influence of coral surface area on microbial community structure while controlling for site and chamber-level variation.

Distance-based redundancy analysis (dbRDA) models were run based on a weighted UniFrac distance ordination for the *Porites lutea* coral-associated sediment dataset. Pearson correlation plots were inspected using *corrplot* package [v.0.95; 72], and collinear variables were removed when r > 0.6 (S1 Fig). Model parameters were adjusted to keep the most significant factors, while concurrently removing any variables with high correlation (VIF score > 5) to reduce the effects of high collinearity in the model. A backward stepwise model was run to further reduce the number of model parameters using the *ordistep* function with 999 permutations (Vegan). Significance was assessed through PERMANOVA (999 permutations) on the *ordistep* output, and dbRDA plots were created in ggplot2 [73]. An additional dbRDA analysis was run using the same model parameters and backward stepwise model, after removing four outliers (13_9a, B6_7a, B6_7b, B6_7c) based on two diagnostic steps. First, relative abundance bar plots show these samples had marked differences in community composition compared with other replicates from the same sites. Second, an unconstrained UniFrac-based ordination indicated these samples were inconsistent with the expected within-site replication (i.e., they were far from their respective site clusters), which can disproportionally influence the model.

Finally, to test for differentially abundant taxa based on continuous variables, we used *DESEq2* package [v.1.38.3;[74]. Separate models were constructed for each environmental covariate (i.e., seawater dissolved oxygen flux, sediment grain size and sediment nutrient variables), using family level taxonomic aggregation. log_2_ Fold Changes were shrinkage-adjusted with the *apeglm* [v.1.20.0; 75] method to improve effect size estimation and control for variance and taxa with adjusted p-values ≤ 0.05 were considered significantly associated with each covariate. The top three taxa with the largest positive and negative log₂ fold changes were plotted to illustrate the most representative bacterial families to each environmental variable.

## Results

### Environmental variables

Gross photosynthetic rates were significantly higher for the seawater contained in the chambers with *Porites lutea* coral-associated sediments (0.52 ± 0.03 g O_2_ m ⁻ ² h ⁻ ¹) compared with seawater in the sediment control chambers (0.32 ± 0.01 g O_2_ m ⁻ ² h ⁻ ¹; χ^2^ = 10.88, p < 0.001; S2A Fig), similarly, respiration rates were also higher for the seawater in the coral-associated chambers (0.24 ± 0.02 g O_2_ m ⁻ ² h ⁻ ¹) compared with sediment control chambers (0.12 ± 0.01 g O_2_ m ⁻ ² h ⁻ ¹; χ^2^ = 16.50, p < 0.001; S2A Fig). There was no significant effect of site for gross photosynthetic rate (χ^2^ = 10.13, p = 0.340) or respiration rate (χ^2^ = 9.69, p = 0.380; S2A Fig).

Across coral-associated sediment samples, gross photosynthesis rates for the seawater ranged from 0.11 to 1.29 g O_2_ m ⁻ ² h ⁻ ¹, while seawater respiration rates varied between 0.01 and 0.53 g O_2_ m ⁻ ² h ⁻ ¹ (S2 Table, S2B Fig), with the photosynthesis to respiration (P:R) ratio ranging between 1.94 to 8.66 (S2 Table, S2C Fig). Total carbon content ranged from 8.59% to 11.45%, total nitrogen from 0.03% to 0.06%, and total organic carbon from 0.08% to 0.29% (S2 Table) while the C:N ratio varied between 1.60 and 6.40 (S2 Table). These coral-associated sediment samples were sand dominated (medium to fine sand), where the gravel fraction (> 2 mm) represented between 0 and 0.08%, sand fraction (2 mm – 63 µm) between 0.81 and 0.99%, and mud fraction (< 63 µm) between 0.01 and 0.19%, with the mean grain size ranging from 0.534 to 2.47 φ (S2 Table).

### Bacterial community composition

The bacterial dataset comprised 141 amplicon libraries (n = 60 sediment samples taken inside incubation chambers with corals and n = 81 sediment samples taken inside incubation chambers with sediment only) of the hypervariable region V3–V4 from the 16S rRNA gene, with an average length of 500 bp. After quality filtering and removing chimaeras and potential contaminants, 8,046,234 total sequences were assigned to the kingdom Bacteria, corresponding to 70,230 unique ASVs, comprising 74 phyla, 179 classes, 555 orders, 1,287 families, and 3,464 genera.

### Comparison between coral-associated and sediment control samples

**Fig 2:**
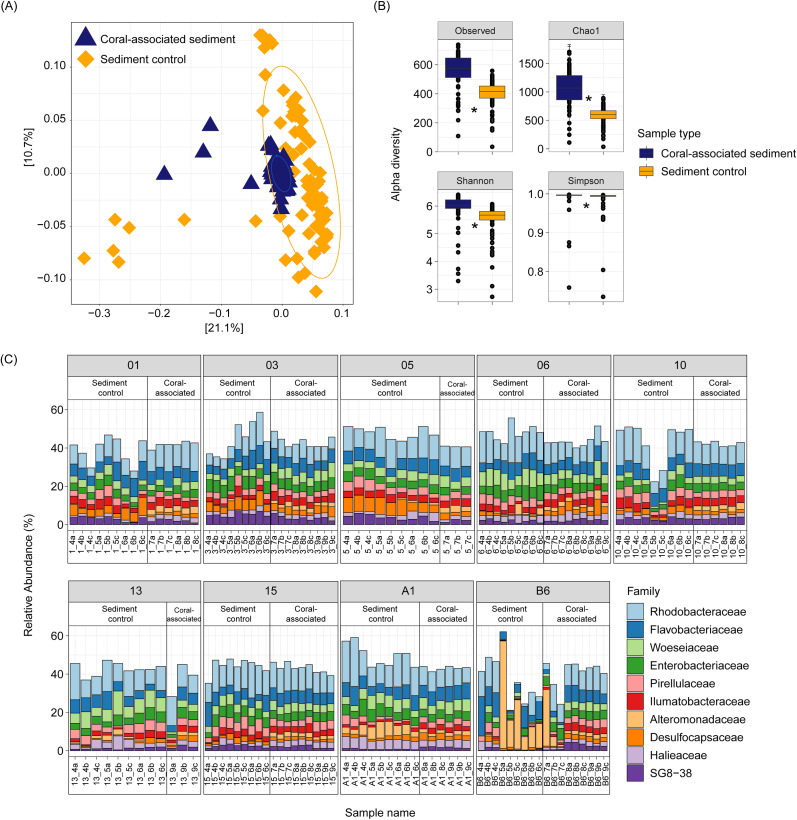
Comparison of coral-associated sediment and sediment control samples. **(A)** Principal coordinates analysis (PCoA) using weighted UniFrac distances for the *Porites lutea* coral (dark blue triangle symbols) and sediment (orange diamond symbols) samples, with the ellipses around the sample type (i.e., coral-associated sediment or sediment control samples). **(B)** Alpha diversity indices for the *Porites lutea* coral (dark blue) and sediment (orange) samples including the observed number of ASVs (Observed), species richness (Chao1), Shannon evenness (Shannon) and Simpson diversity (Simpson). Significant difference indicated by asterisk **(C)** Relative abundance of the top ten families in each replicate for the sediment and *Porites lutea* coral samples, across each site.

Principal coordinates analysis (PCoA; weighted UniFrac) showed coral-associated and sediment-only bacterial communities partially overlapped (Fig 2A), with the sediment samples showing greater dispersion (within-group variability) than coral-associated sediment samples (axis 1: 21.1% variance and axis 2: 10.7% variance; Fig 2A). PERMANOVA indicated that bacterial community composition differed significantly between coral-associated and control sediments (adonis2: F = 16.60, R² = 0.087, p < 0.0001; Fig 2A) after accounting for site effects. Site-level variation also explained a substantial portion of community differences (F = 5.38, R² = 0.225, p < 0.0001). When permutations were constrained within sites, the difference between coral-associated and control sediments remained significant (F = 13.88, R² = 0.091, p < 0.0001; S3A Fig), confirming that this pattern was consistent within sites rather than driven by among-site variation. However, multivariate dispersion differed between sample type (betadisper: F = 51.47, p < 0.0001), indicating that sediment control samples exhibited greater variability in community composition than coral-associate sediments.

Alpha diversity indices comparing the coral-associated and sediment only samples revealed coral-associated samples were significantly higher for all metrics (the observed number of ASV’s (X² = 50.373, p < 0.001), Chao1 (X² = 65.173, p < 0.001), Shannon (X² = 45.465, p < 0.001), Simpson (X² = 36.232, p < 0.001; S1 Table, Fig 2B)).

At the family level, the most abundant families for both the coral-associated and sediment-only samples were Rhodobacteraceae, Flavobacteriaceae, and Woeseiaceae. In the coral-associated samples, Rhodobacteraceae comprised 10.56% ± 0.29 SE average relative read abundance, followed by Flavobacteriaceae (6.24% ± 0.17) and Woeseiaceae (4.50% ± 0.18; Fig 2C), while the sediment only samples were dominated by Flavobacteriaceae (6.69% ± 0.30), followed by Rhodobacteraceae (5.97% ± 0.54) then Woeseiaceae (4.49% ± 0.26; Fig 2C).

Differential abundance analysis of relative read abundance (RRA) identified 283 bacterial families with significant differences in abundance between the coral-associated sediment versus sediment control samples. In the coral-associated sediment, there was a 24.64 ± 1.27 log_2_ FC increase in relative read abundance of Flavobacteriaceae compared with the sediment controls (p < 0.001; S3B Fig). In contrast, Sphingomonadaceae was significantly enriched in the sediment control samples compared with the coral-associated samples (27.21 ± 6.94 log_2_ FC, p < 0.001; S3B Fig).

Three bacterial families showed significant responses to gross photosynthesis and respiration rates. Woeseiaceae, Pirellulaceae and Streptomycetaceae were significantly enriched with increasing gross photosynthesis and respiration (all p < 0.002; S3 Table, S4 Fig). There was a negative association for Rhodobacteraceae with a 6.8 log_2_ FC decrease in relative read abundance (RRA) with increasing total carbon content (p < 0.001; S3 Table, S4 Fig), whereas there was a positive association for total nitrogen, with a 9.07 log_2_ FC increase in abundance with increasing total nitrogen (S3 Table, S4 Fig). We also found an 11.32 log_2_ FC decrease (p < 0.001; S3 Table, S4 Fig) in the RRA of Alteromonadaceae with increasing total nitrogen, but a 16.35 log_2_ FC increase with increasing C:N ratio (p < 0.001; S3 Table, S4 Fig). For the sediment grain size variables, there was a negative association for Alteromonadaceae) with mean grain size and gravel content (17.55 and 8.58 log_2_ FC decrease, respectively, both p < 0.001; S3 Table, S4 Fig), but a positive association with the sand fraction with a 26.33 log_2_ FC increase (p < 0.001; S3 Table, S4 Fig). Additionally, family GCA-2696645 was enriched with increasing sand fraction (24.19 log_2_ FC increase), with a 28.26 log_2_ FC decrease in the mud fraction (both p < 0.001; S3 Table, S4 Fig). Finally, we also found Enterobacteriaceae was negatively associated with increasing mean grain size (14.39 log_2_ FC decrease, p < 0.001) and gravel content (6.12 log_2_ FC decrease, p < 0.001; S3 Table, S4 Fig).

We found four shared families across the coral-associated and sediment only samples, with the largest abundance of Rhodobacteraceae (relative read abundance (RRA): 11.33 ± 0.35%), followed by Flavobacteriaceae (6.50 ± 0.19% RRA). There were 30 families of bacterial taxa that were found in all the coral-associated samples (representing 58% of total relative abundance), of these there were 26 exclusive to the coral-associated samples with four shared with the sediment controls (S3C Fig). There were no unique families for the sediment controls (S3C Fig).

Following the above analyses highlighting the differences between the *Porites lutea* coral-associated and sediment control samples, we present the remaining analysis with the coral-associated chambers only to further explore variation and environmental drivers of community structure.

### Bacterial community structure of the porites lutea coral-associated sediment

**Fig 3:**
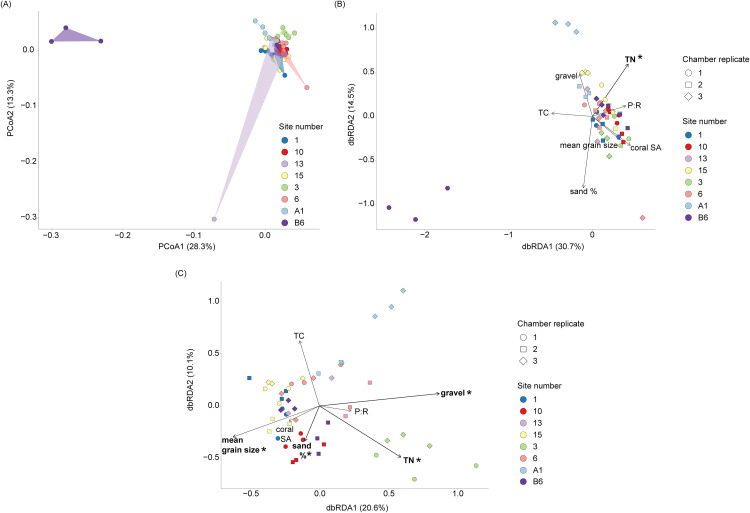
Patterns and predictors of sediment microbial community structure. **(A)** Principal coordinates analysis (PCoA) of microbial community structure using weighted UniFrac distances. Each point represents a coral-associated chamber, coloured by site. Axes indicate the first two principal coordinates, with percentages reflecting the proportion of variance explained. **(B)** dbRDA for the coral-associated sediment samples with the environmental parameters displayed as vectors (black lines) driving the community structure. Asterisk (TN content) indicates statistical significance (p < 0.05) derived from a dbRDA back wise step model. **(C)** dbRDA for the coral-associated samples after removing four outlier samples, with the environmental parameters as vectors (black lines) driving the community structure and asterisks indicating statistical significance (p < 0.05) derived from a dbRDA back wise step model.

To examine the coral-associated sediment community structure, we first performed a principal coordinate analysis (PCoA) based on weighted UniFrac distances and found clear separation of site (B6), where the first two axes explained 28.3% and 13.3% of the total variation (Fig 3A). PERMANOVA revealed coral surface area had a small but significant effect on bacterial community structure (R² = 0.027, F = 2.06, p = 0.027), whereas nested site and chamber replicate factors accounted for a larger proportion of variation (R² = 0.179, F = 1.94, p < 0.001). Together this suggests that microbial communities are predominantly influenced by site effects.

Distance-based redundancy analysis (dbRDA) revealed clear differentiation between microbial communities from sites B6 and A1, with dbRDA1 and dbRDA2 explaining 31.2% and 14.2% of total variation, respectively (Fig. 3B). There was a significant effect for total nitrogen (F = 2.17, p = 0.024, Table 1, Fig 3B), explaining 4.1% of the total variation in microbial communities (adjusted R^2^ = 0.041). After removing four outlier samples, dbRDA revealed gravel content (F = 5.73, p = 0.001), sand % (F = 2.74, p = 0.004), mean grain size (F = 2.77, p = 0.002) and total nitrogen (F = 3.69, p = 0.001; Table 1, Fig 3C) significantly influenced microbial community structure, collectively explaining 18.2% of the total variation (adjusted R^2^ = 0.182).

**Table 1:** Summary of distance-based Redundancy Analysis (dbRDA) results following backward model selection using *ordistep*. The table presents the environmental variables retained in the final model, their degrees of freedom (df), sum of squares, F statistic and significance level (p value) for (A) all *Porites lutea* coral-associated sediment samples and (B) a subset of the *Potites lutea* coral-associated sediment samples with four outlier replicates removed.

	Variable	Df	Sum of Squares	F value	P value
A)	Total nitrogen	1	0.03	2.17	0.024
	Residual	51	0.72		
B)	Gravel content	1	0.03	5.73	0.001
	Sand %	1	0.02	2.74	0.004
	Mean grain size	1	0.02	2.77	0.002
	Total nitrogen	1	0.02	3.69	0.001
	Residual	45	0.27		

### Differential abundance of bacterial taxa in response to environmental covariates

Differential abundance testing of relative read abundance (RRA) data identified the number of bacterial families and the magnitude of significant responses among environmental variables. For gross photosynthesis and respiration rates, there were three notable bacterial families that significantly responded to these covariates. Specifically, Woeseiaceae, Pirellulaceae and Streptomycetaceae were significantly enriched with increasing gross photosynthesis and respiration (all p < 0.002; S3 Table, S4 Fig). There was a negative association for Rhodobacteraceae with a 6.8 log_2_ FC decrease in relative read abundance (RRA) with increasing total carbon content (p < 0.001), whereas there was a positive association for total nitrogen, with a 9.07 log_2_ FC increase in abundance with increasing total nitrogen (S3 Table, S4 Fig). We also found an 11.32 log_2_ FC decrease (p < 0.001) in the RRA of Alteromonadaceae with increasing total nitrogen, but a 16.35 log_2_ FC increase with increasing C:N ratio (p < 0.001). For the sediment grain size variables, there was a negative association for Alteromonadaceae with mean grain size and gravel content (17.55 and 8.58 log_2_ FC decrease, respectively, both p < 0.001; S3 Table, S4 Fig), but a positive association with the sand fraction with a 26.33 log_2_ FC increase (p < 0.001; S3 Table, S4 Fig). Additionally, GCA-2696645 was enriched with increasing sand fraction (24.19 log_2_ FC increase), with a 28.26 log_2_ FC decrease in the mud fraction (both p < 0.001; S3 Table, S4 Fig). Finally, we also found Enterobacteriaceae was negatively associated with increasing mean grain size (14.39 log_2_ FC decrease, p < 0.001) and gravel content (6.12 log_2_ FC decrease, p < 0.001; S3 Table, S4 Fig).

## Discussion

To accurately monitor the health of coral reefs, it is crucial to understand how each component of the reef contributes to the ecological stability and function of the entire coral reef ecosystem [1]. Despite microbial abundances in coral reef sediments being several orders of magnitude higher than in the overlying seawater (~1–2 × 10⁹ cells cm ⁻ ² in sediments compared with ~3–9 × 10⁵ cells mL ⁻ ¹ in nutrient-poor reef waters [16,76]), bacterial communities in marine sediments remain comparatively understudied. Sediment bacteria significantly contribute to the ecological stability and function of coral reef ecosystems and serve as good indicators of environmental perturbations [23,24]. In addition, studies suggest that corals may interact directly with sediment bacteria, for example, by acquiring bacteria that are beneficial to the coral or pathogenic [20,22,41]. In this study, we provide a detailed comparison of the metabolic activity, sediment properties and bacterial communities of sediments in close proximity to healthy *Porites lutea* corals (i.e., coral-associated sediment) and sediment controls within the main lagoon of OTI reef. Our results show the seawater contained within the chambers with coral-associated sediments exhibit higher gross photosynthetic and respiration rates, as well as higher bacterial richness and diversity, compared to the seawater in the sediment control chambers. Bacterial community composition differed between sample types, with the coral-associated sediments showing a taxonomically distinct bacterial community. Within the coral-associated sediment, total nitrogen content contributed to variation in microbial structure but sediment physical properties also exerted a strong influence. Together, these findings indicate that microbial assemblages in reef sediments are primarily shaped by sediment physicochemical properties and benthic metabolic flux, rather than coral trophic state alone.

### Enhanced metabolic activity in coral-associated sediments

The higher gross photosynthesis and respiration rates in the seawater contained in the *P. lutea* coral-associated chambers, compared with the seawater from the sediment only control chambers, suggests that coral proximity increases benthic metabolic activity. This may be due to increased inputs of organic matter close to coral colonies, as the result of corals’ shedding mucus. Coral mucus dissolves in seawater where it fuels the microbial loop or can sink to be degraded by sediment microbial communities, which results in the recycling of essential nutrients and energy back into the reef system [27]. This is also supported by the distinct bacterial communities found in the coral-associated versus the sediment control chambers (discussed in greater depth below), because coral mucus creates an ideal environment for microbial growth and represents one of the largest sources of organic matter in the coral reef ecosystem [28]. This is further supported by the dominance of medium to fine sands in our study, as permeable sediments typically facilitate higher rates of interstitial water exchange and microbial turnover [77,78]. Further laboratory and in situ studies would help clarify how coral-derived organic matter, sediment permeability, and microbial communities interact to regulate benthic metabolism and nutrient cycling.

### Distinct bacterial community structure between coral-associated and control sediments

Bacterial communities differed significantly between coral-associated sediments and sediment-only controls, with the coral-associated samples exhibiting smaller within group variability and higher alpha diversity indices compared with the sediment-only controls. This suggests the *P. lutea* coral promotes a more biodiverse and compositionally consistent bacterial assemblage in the closely-associated sediment. This compliments the enhanced metabolic activity in the coral-associated chambers, where the coral exudates, mucus and organic matter create a nutrient enriched microenvironment in the closely-associated sediment that supports a broader range of microbial taxa, as discussed above. Furthermore, we found distinct bacterial communities in the coral-associated versus the sediment control samples, consistent with previous work describing niche specialisation by marine microbes [14,79]. Niche partitioning or variability in the microbial community composition and metabolic function, has been identified for coral, sediment and seawater [14,79]. Coral colonies have previously been shown to host diverse and species-specific microbial communities [80,81] that are distinct from those in adjacent seawater [14,81–83], and individual sand grains have been reported to harbour several thousand bacteria each [77]. Additionally, microbes associated with other benthic organisms on coral reefs have shown patterns of host specificity – for example, sponges harbour diverse and abundant microbial communities, which generally exhibit species specificity regardless of geography [84].

The dominant families Rhodobacteraceae, Flavobacteriaceae, and Woeseiaceae were shared across sample types, yet their relative abundance differed for both sample groups, highlighting microbial niche differentiation between coral-associated and adjacent sediment control samples. The contribution of Rhodobacteraceae as the dominant shared family across coral-associated and sediment control samples is notable, as this group is known for their role in organic matter degradation and sulfur cycling [85]. Despite no exclusive taxa for the sediment controls, compared with the 26 unique families for the coral-associated samples, we found the influence of the coral provides a distinct and diverse microbial assemblage.

The two most abundant families present across both sample types were Rhodobacteraceae and Flavobacteriaceae. The family Rhodobacteraceae, within the class Alphaproteobacteria, are widespread in marine habitats and are typically considered ecological generalists [85,86]. Bacteria of this family are categorised as extreme and moderate oligotrophs [87], and have relatively flexible requirements for carbon and energy metabolism, which may allow them to respond to a diverse range of environmental conditions [88]. *HIMB11* is a member of the family Rhodobacteraceae, and despite its comparatively more streamlined and smaller genome size (indicating a more specialised lifestyle [89]), it has been found to be capable of degrading dimethylsulfoniopropionate [90], a major source of organic sulfur found in significant concentrations within corals, with the ability to harvest energy from carbon monoxide [91] and light [92].

The family Flavobacteriaceae are found in a broad range of marine and non-marine habitats and are key heterotrophic bacteria in marine ecosystems commonly found in sediment [93,94]. Regardless of their habitats, Flavobacteriaceae are generally considered as specialists for the degradation of complex organic matter, including polysaccharides [93], with these degradation products subsequently made available for other microorganisms [93]. The genus *Aequorivita* from Flavobacteriaceae plays a functional role in nutrient cycling and the breakdown of organic matter, with some strains associated with antimicrobial activity [95].

Microbial populations that are consistently present across assemblages [96], are thought to contribute to the overall health and metabolic functioning of their hosts [97]. Consistent with the overall taxonomic patterns described above, Rhodobacteraceae and Flavobacteraceae were consistently present in both the coral-associated sediment and sediment control (S3b Fig). Previous work has identified Rhodobacteraceae and Endozoicomonadaceae as key families associated with *P. lutea* [98,99]. Although Endozoicomonadaceae was not consistently present in the coral-associated sediment in our study, it was observed at low overall total relative abundance (0.04%) across these samples. The absence of Endozoicomonadaceae from the sediment only controls, supports previous work indicating coral host specificity [100], and its low abundance in coral-associated sediment may reflect localised seeding from the coral microbiome, potentially via mucus shedding. This further suggests corals can influence adjacent sediment communities, yet the spatial extent and ecological significance of this influence remain important areas for further investigation.

Although our approach in this study enabled us to identify unique bacterial communities for the coral-associated and sediment control samples, we acknowledge there may be a contribution from the associated communities in the overlying and interstitial seawater, which was not described in this study. Further, it’s also worth noting that the difference between replicates at each site could be due to the possibility of sampling clonal colonies at each site, and without genetic testing to confirm coral heads weren’t from the same parent colonies we are unable to confirm variability due to host genotype [101]. This may be an issue because differences in mucus production have been described between and within coral species [102], therefore genotypes potentially have different rates of mucus shedding.

### Environmental drivers of coral-associated microbial community structure

Total nitrogen content was a key environmental driver explaining variation in coral-associated sediment bacterial assemblages, consistent with previous studies showing strong associations between microbial communities and sediment habitat properties [103]. While total nitrogen does not represent bioavailable nitrogen, it may covary with sediment characteristics such as permeability and organic matter content. Sediment permeability can influence nitrogen transformations and exchange with the overlying water column and has been shown to be positively associated with the bioactivity of calcareous sediments [30,33]. In permeable sediments, enhanced denitrification can lead to the loss of bioavailable fixed nitrogen from the sediment to the water column [29,30]. Furthermore, elevated total nitrogen may reflect increased organic matter content, which could indirectly influence the distribution of putative nitrogen-fixing bacterial taxa; however, because nitrogen speciation or production rates were not measured in the current study, these interpretations remain speculative. This aligns with previous work on marine sediments, where dose-dependent effects of total nitrogen were linked to shifts in microbial composition [104]. Although total nitrogen content only explained ~4% of community variation in our study, complex benthic systems such as the marine sediments surrounding coral colonies are typically shaped by multiple, interacting gradients [105,106]. This was evident from the increased explanatory power observed (~18% of the community variation) when sediment physical properties were included (after removing four outliers), highlighting that both sediment nutrients and physical characteristics influence microbial assemblages.

### Differential taxonomic responses to environmental variables

Differential abundance analyses revealed key bacterial families exhibited distinct responses to environmental gradients, highlighting the ecological versatility of sediment microbial assemblages to changes in nutrient availability, productivity, and sediment properties (S3 Table, S4 Fig). The enrichment of Woeseiaceae, Pirellulaceae, and Streptomycetaceae with increasing gross photosynthesis and respiration within the chamber suggests that these taxa are metabolically linked to elevated organic carbon inputs and oxygen fluxes. Members of Woeseiaceae and Pirellulaceae have previously been reported as dominant families in marine sediment, including in Caribbean reef systems [41], and are often associated with oligotrophic marine sediments playing central roles in organic matter degradation and nitrogen cycling. Similarly, Streptomycetaceae are well-known decomposers in both marine and terrestrial environments, that increase in abundance under higher carbon turnover and aerobic conditions [107].

Nutrient related variables further structured the microbial assemblages, with Rhodobacteraceae and Alteromonadaceae exhibiting opposing responses to C:N ratios. The enrichment of Rhodobacteraceae under increasing nitrogen, but their decline with higher carbon, suggests divergent nutrient acquisition strategies that align with their known photoheterotrophic and denitrifying metabolisms [85]. In contrast, Alteromonadaceae increased under high C:N ratios but declined under elevated nitrogen, consistent with a copiotrophic, carbon-responsive lifestyle [108]. These patterns are supported by findings from deep-sea [108] and coral reef sediments [109] where the genus *Pseudoalteromonas* within family Alteromonadaceae thrives under carbon-rich, nitrogen-limited conditions. Despite the common occurrence of *Pseudoalteromonas* in sediments, little information exists on how they thrive in marine sediments [but see Qin *et al.*, 107].

Sediment grain-size variables also exerted a strong influence on community structure, by influencing organic matter retention, oxygen penetration, permeability and the physical surface area available for microbial colonisation, all of which shape microbial habitats environment [110]. The decrease in Enterobacteriaceae with increasing grain size and gravel content suggests these facultative anaerobes prefer finer, organic-rich sediments, consistent with patterns in lake sediments [111]. Similarly, Alteromonadaceae increased with higher sand fractions and decreased in gravel-dominated fractions, highlighting their affinity for oxic, permeable substrates. Our findings also align with work in seagrass sediments, showing sediment physical structure is a key determinant of microbial community composition [112]. Taken together, the interaction between sediment physical properties, productivity and nutrient concentrations in shaping microbial communities is complex and requires further investigation to better understand how coral reef sediments regulate microbial diversity and ecosystem functioning.

## Conclusion

This study provides a detailed characterisation of sediment bacterial communities adjacent to healthy *Porites lutea* colonies in a pristine reef lagoon. Despite the close proximity to corals, sediment bacterial communities are predominantly shaped by local physicochemical gradients rather than by coral trophic traits alone. Coral-associated sediments showed enhanced metabolic activity of the seawater contained within the chamber, higher taxonomic richness and distinct microbial communities, yet these patterns were best explained by nutrient availability, particularly total nitrogen and sediment physical properties. By integrating microbial composition with measures of benthic metabolism, sediment properties and nutrient availability, we provide insight into how reef sediment structure and nutrients influence reef function. These findings establish a microbial baseline for detecting future shifts in sediment community structure in response to coral health decline and broader environmental perturbations.

## Supporting information

S1 Table Alpha diversity metrics for the *Porites lutea* coral-associated and sediment only control samples, including; the observed number of ASV’s, Chao1 richness, Shannon diversity, and Simpson diversity. Showing the number of samples (n), minimum (min), maximum (max), mean and standard error (SE).
(PDF)

S2 Table Summary table for the environmental variables for the *Porites lutea* coral-associated samples. Missing data for one chamber at site 03 (03_8).
(PDF)

S3 Table Results of differential abundance of bacterial taxa across environmental covariates, identified using DESEq2 for the *Porites lutea* coral-associated samples. DESEq2 was run for each covariate in a single-term model, and log_2_ Fold Changes were shrunk using the *apeglm* method. The table shows the top three positive and negative significantly differentially abundant taxa with adjusted p values, merged with taxonomic annotations to genus, as well as the respective ASV. Positive log_2_ Fold Changes indicate higher abundance with increasing covariate values, while negative values indicate decreasing abundance. Continuous environmental covariates were Z-scaled prior to analysis. Scientific numbers used for log_2_ Fold Changes to show small numbers to 2 decimal places.
(PDF)

S1 Fig Correlation plots for the *Porites lutea* coral-associated sediment samples showing pairwise Pearson correlations among variables for the (A) dissolved oxygen flux variables (green), (B) sediment grain size variables (orange) and (C) sediment nutrients variables (blue). Colour intensity represents the strength and direction of correlation Correlated variables were removed for dbRDA analyses.
(PDF)

S2 Fig Seawater dissolved oxygen flux (g m^-2^ h^-1^) measured inside the incubation chambers for (A) the *Porites lutea* coral-associated and sediment control samples, displayed as averages for each site and (B) the coral-associated samples for each replicate. Both representing gross primary production rate (green bars), net primary production (red bars) and respiration rate (yellow bars). The net primary production was calculated by subtracting the respiration rate from the gross primary production rate. (C) The photosynthesis to respiration ratio was calculated for *Porites lutea* and used as a proxy for overall coral health. The red dashed line represents a P:R ratio of 1, where P:R > 1 indicates that autotrophic activity is higher than heterotrophic activity, meaning the system is a net producer of organic matter, and conversely when the P:R < 1 heterotrophic activity predominates over autotrophy.
(PDF)

S3 Fig Comparison of *Porites lutea* coral-associated sediment and sediment control samples. (A) Principal coordinates analysis (PCoA) using weighted UniFrac distances for the *Porites lutea* coral (triangle symbols) and sediment samples (circle symbols) for each site, with the ellipses around the sample type (i.e., coral-associated sediment or sediment control samples). (B) Top bacterial taxa (adjusted p ≤ 0.05) identified using DESeq2 differential abundance for the coral-associated sediment and sediment control samples. Points represent individual genera (annotated with family, genus and ASV ID), coloured by bacterial class, with positive log_2_ Fold Changes indicating these taxa were in higher abundance in the sediment control samples, while negative log₂ fold changes indicate taxa higher in abundance for the coral-associated sediment samples. Horizontal dashed line at zero indicates no change in abundance. (C) Core microbial community for the coral-associated sediment and sediment control samples, run at the taxonomic level of family. Insert (c) shows a Venn diagram for the coral-associated sediment and sediment controls with the numbers inside the sections representing the number of unique and shared families.
(PDF)

S4 Fig Top bacterial taxa responding to environmental covariates (adjusted p ≤ 0.05) identified using DESeq2 differential abundance. Points represent individual families (annotated with genus and ASV ID), coloured by bacterial class, with positive and negative log₂ fold changes indicating significantly higher or lower abundance with increasing covariate values, respectively. Horizontal dashed line at zero indicates no change in abundance.
(PDF)

## References

[1] Dong X, Lan H, Huang L, Zhang H, Lin X, Weng S, et al. Metagenomic views of microbial communities in sand sediments associated with coral reefs. Microb Ecol. 2023;85(2):465–77. doi: 10.1007/s00248-021-01957-8 35113183

[2] Glasl B, Bourne DG, Frade PR, Webster NS. Establishing microbial baselines to identify indicators of coral reef health. Microbiol Aust. 2018;39:42. doi: 10.1071/ma18011

[3] Great Barrier Reef Marine Park Authority (GBRMPA. Priority monitoring gaps prospectus: Reef 2050 Integrated Monitoring and Reporting Program. Australian Government; 2021. https://hdl.handle.net/11017/3788

[4] Great Barrier Reef Marine Park Authority (GBRMPA). Chapter 2: Biodiversity. Great Barrier Reef Outlook Report 2019. Great Barrier Reef Marine Park Authority; 2019. 15–46. http://hdl.handle.net/11017/3474

[5] Bellwood DR. Production and reworking of sediment by parrotfishes (family Scaridae) on the Great Barrier Reef, Australia. Mar Biol. 1996;125:795–800. doi: 10.1007/bf00349262

[6] Yarlett RT, Perry CT, Wilson RW. Quantifying production rates and size fractions of parrotfish-derived sediment: A key functional role on Maldivian coral reefs. Ecol Evol. 2021;11(22):16250–65. doi: 10.1002/ece3.8306 34824825 PMC8601892

[7] Bosence D. Biogenic carbonate production in Florida Bay. Bull Mar Sci. 1989;44:419–33.

[8] Perry CT, Taylor KG, Machent PG. Temporal shifts in reef lagoon sediment composition, Discovery Bay, Jamaica. Estuarine, Coastal and Shelf Science. 2006;67:133–44. doi: 10.1016/j.ecss.2005.11.031

[9] Wizemann A, Mann T, Klicpera A, Westphal H. Microstructural analyses of sedimentary Halimeda segments from the Spermonde Archipelago (SW Sulawesi, Indonesia): A new indicator for sediment transport in tropical reef islands?. Facies. 2015;61(2). doi: 10.1007/s10347-015-0429-5

[10] Perry CT, Kench PS, Smithers SG, Yamano H, O’Leary M, Gulliver P. Time scales and modes of reef lagoon infilling in the Maldives and controls on the onset of reef island formation. Geology. 2013;41(10):1111–4. doi: 10.1130/g34690.1

[11] Kappelmann Y, Westphal H, Kneer D, Wu HC, Wizemann A, Jompa J, et al. Fluctuating sea-level and reversing Monsoon winds drive Holocene lagoon infill in Southeast Asia. Sci Rep. 2023;13(1):5042. doi: 10.1038/s41598-023-31976-z 36977704 PMC10050433

[12] Uthicke S, McGuire K. Bacterial communities in Great Barrier Reef calcareous sediments: Contrasting 16S rDNA libraries from nearshore and outer shelf reefs. Estuarine, Coastal and Shelf Science. 2007;72(1–2):188–200. doi: 10.1016/j.ecss.2006.10.017

[13] Schöttner S, Pfitzner B, Grünke S, Rasheed M, Wild C, Ramette A. Drivers of bacterial diversity dynamics in permeable carbonate and silicate coral reef sands from the Red Sea. Environ Microbiol. 2011;13(7):1815–26. doi: 10.1111/j.1462-2920.2011.02494.x 21554515 PMC3207121

[14] Glasl B, Bourne DG, Frade PR, Thomas T, Schaffelke B, Webster NS. Microbial indicators of environmental perturbations in coral reef ecosystems. Microbiome. 2019;7(1):94. doi: 10.1186/s40168-019-0705-7 31227022 PMC6588946

[15] Hoshino T, Doi H, Uramoto G-I, Wörmer L, Adhikari RR, Xiao N, et al. Global diversity of microbial communities in marine sediment. Proc Natl Acad Sci U S A. 2020;117(44):27587–97. doi: 10.1073/pnas.1919139117 33077589 PMC7959581

[16] Wild C, Laforsch C, Huettel M. Detection and enumeration of microbial cells within highly porous calcareous reef sands. Mar Freshw Res. 2006;57:415. doi: 10.1071/mf05205

[17] Schöttner S, Wild C, Hoffmann F, Boetius A, Ramette A. Spatial scales of bacterial diversity in cold-water coral reef ecosystems. PLoS One. 2012;7(3):e32093. doi: 10.1371/journal.pone.0032093 22403625 PMC3293894

[18] Chavanich S, Kusdianto H, Kullapanich C, Jandang S, Wongsawaeng D, Ouazzani J. Microbiomes of healthy and bleached corals during a 2016 thermal bleaching event in the andaman sea of Thailand. Front Mar Sci. 2022;9. doi: 10.3389/fmars.2022.763421

[19] Aigars J. The role of sediments in the biogeochemical cycles of nutrients in the Gulf of Riga. PhD, Stockholm University. 2001. https://dspace.lu.lv/handle/7/376

[20] Carlos C, Torres TT, Ottoboni LMM. Bacterial communities and species-specific associations with the mucus of Brazilian coral species. Sci Rep. 2013;3:1624. doi: 10.1038/srep01624 23567936 PMC3620669

[21] Nitschke M. The free-living Symbiodinium reservoir and scleractinian coral symbiont acquisition. PhD, The University of Queensland. 2015. 10.14264/uql.2015.687

[22] Glasl B, Herndl GJ, Frade PR. The microbiome of coral surface mucus has a key role in mediating holobiont health and survival upon disturbance. ISME J. 2016;10(9):2280–92. doi: 10.1038/ismej.2016.9 26953605 PMC4989324

[23] Great Barrier Reef Marine Park Authority (GBRMPA). Chapter 3: Ecosystem Health. Great Barrier Reef Outlook Report 2019. Great Barrier Reef Marine Park Authority; 2019. 47–82. http://hdl.handle.net/11017/3474

[24] Pernice M, Raina J-B, Rädecker N, Cárdenas A, Pogoreutz C, Voolstra CR. Down to the bone: The role of overlooked endolithic microbiomes in reef coral health. ISME J. 2020;14(2):325–34. doi: 10.1038/s41396-019-0548-z 31690886 PMC6976677

[25] Hamamoto K, Mizuyama M, Nishijima M, Maeda A, Gibu K, Poliseno A, et al. Diversity, composition and potential roles of sedimentary microbial communities in different coastal substrates around subtropical Okinawa Island, Japan. Environ Microbiome. 2024;19(1):54. doi: 10.1186/s40793-024-00594-1 39080706 PMC11290285

[26] Rusch A, Hannides AK, Gaidos E. Diverse communities of active Bacteria and Archaea along oxygen gradients in coral reef sediments. Coral Reefs. 2008;28(1):15–26. doi: 10.1007/s00338-008-0427-y

[27] Song J, Hwang J, Kang I, Cho J-C. A sulfate-reducing bacterial genus, Desulfosediminicola gen. nov., comprising two novel species cultivated from tidal-flat sediments. Sci Rep. 2021;11(1):19978. doi: 10.1038/s41598-021-99469-5 34620953 PMC8497536

[28] Wild C, Huettel M, Klueter A, Kremb SG, Rasheed MYM, Jørgensen BB. Coral mucus functions as an energy carrier and particle trap in the reef ecosystem. Nature. 2004;428(6978):66–70. doi: 10.1038/nature02344 14999280

[29] Bednarz V, van Hoytema N, Cardini U, Naumann M, Al-Rshaidat M, Wild C. Dinitrogen fixation and primary productivity by carbonate and silicate reef sand communities of the Northern Red Sea. Mar Ecol Prog Ser. 2015;527:47–57. doi: 10.3354/meps11224

[30] El-Khaled YC, Roth F, Rädecker N, Tilstra A, Karcher DB, Kürten B, et al. Nitrogen fixation and denitrification activity differ between coral- and algae-dominated Red Sea reefs. Sci Rep. 2021;11(1):11820. doi: 10.1038/s41598-021-90204-8 34083565 PMC8175748

[31] Elser JJ, Bracken MES, Cleland EE, Gruner DS, Harpole WS, Hillebrand H, et al. Global analysis of nitrogen and phosphorus limitation of primary producers in freshwater, marine and terrestrial ecosystems. Ecol Lett. 2007;10(12):1135–42. doi: 10.1111/j.1461-0248.2007.01113.x 17922835

[32] Cardini U, Bednarz VN, Foster RA, Wild C. Benthic N2 fixation in coral reefs and the potential effects of human-induced environmental change. Ecol Evol. 2014;4(9):1706–27. doi: 10.1002/ece3.1050 24967086 PMC4063469

[33] Capone D, Dunham S, Horrigan S, Duguay L. Microbial nitrogen transformations in unconsolidated coral reef sediments. Mar Ecol Prog Ser. 1992;80:75–88. doi: 10.3354/meps080075

[34] Thompson JR, Rivera HE, Closek CJ, Medina M. Microbes in the coral holobiont: Partners through evolution, development, and ecological interactions. Front Cell Infect Microbiol. 2015;4:176. doi: 10.3389/fcimb.2014.00176 25621279 PMC4286716

[35] Zhang Y, Ling J, Yang Q, Wen C, Yan Q, Sun H, et al. The functional gene composition and metabolic potential of coral-associated microbial communities. Sci Rep. 2015;5:16191. doi: 10.1038/srep16191 26536917 PMC4633650

[36] Littman R, Willis BL, Bourne DG. Metagenomic analysis of the coral holobiont during a natural bleaching event on the Great Barrier Reef. Environ Microbiol Rep. 2011;3(6):651–60. doi: 10.1111/j.1758-2229.2010.00234.x 23761353

[37] Gardner SG, Raina J-B, Nitschke MR, Nielsen DA, Stat M, Motti CA, et al. A multi-trait systems approach reveals a response cascade to bleaching in corals. BMC Biol. 2017;15(1):117. doi: 10.1186/s12915-017-0459-2 29216891 PMC5719617

[38] de Voogd NJ, Cleary DFR, Polónia ARM, Gomes NCM. Bacterial community composition and predicted functional ecology of sponges, sediment and seawater from the thousand islands reef complex, West Java, Indonesia. FEMS Microbiol Ecol. 2015;91(4):fiv019. doi: 10.1093/femsec/fiv019 25764467

[39] Cleary DFR, Polónia ARM, Becking LE, de Voogd NJ, Gomes H. Compositional analysis of bacterial communities in seawater, sediment, and sponges in the Misool coral reef system, Indonesia. Mar Biodivers. 2017;48:1889–901. doi: 10.1007/s12526-017-0697-0

[40] León-Zayas R, McCargar M, Drew JA, Biddle JF. Microbiomes of fish, sediment and seagrass suggest connectivity of coral reef microbial populations. PeerJ. 2020;8:e10026. doi: 10.7717/peerj.10026 33005496 PMC7513772

[41] Hernández-Zulueta J, Díaz-Pérez L, Echeverría-Vega A, Nava-Martínez GG, García-Salgado MÁ, Rodríguez-Zaragoza FA. An update of knowledge of the bacterial assemblages associated with the mexican caribbean corals acropora palmata, orbicella faveolata, and porites porites. Diversity. 2023;15(9):964. doi: 10.3390/d15090964

[42] Rosales SM, Clark AS, Huebner LK, Ruzicka RR, Muller EM. Rhodobacterales and rhizobiales are associated with stony coral tissue loss disease and its suspected sources of transmission. Front Microbiol. 2020;11:681. doi: 10.3389/fmicb.2020.00681 32425901 PMC7212369

[43] Studivan MS, Rossin AM, Rubin E, Soderberg N, Holstein DM, Enochs IC. Reef sediments can act as a stony coral tissue loss disease vector. Frontiers in Marine Science. 2022;8. doi: 10.3389/fmars.2021.815698

[44] Bythell JC, Wild C. Biology and ecology of coral mucus release. J Exp Mar Biol Ecol. 2011;408:88–93. doi: 10.1016/j.jembe.2011.07.028

[45] Roth F, Wild C, Carvalho S, Rädecker N, Voolstra CR, Kürten B, et al. An in situ approach for measuring biogeochemical fluxes in structurally complex benthic communities. Methods Ecol Evol. 2019;10(5):712–25. doi: 10.1111/2041-210x.13151

[46] Ferrari R, McKinnon D, He H, Smith R, Corke P, González-Rivero M, et al. Quantifying multiscale habitat structural complexity: A cost-effective framework for underwater 3D modelling. Remote Sensing. 2016;8(2):113. doi: 10.3390/rs8020113

[47] Harianto J, Carey N, Byrne M. RespR—An R package for the manipulation and analysis of respirometry data. Methods Ecol Evol. 2019;10(6):912–20. doi: 10.1111/2041-210x.13162

[48] R Core Team. R: A language and environment for statistical computing. Vienna, Austria: R Foundation for Statistical Computing. 2021.

[49] Bidwell RGS. Photosynthesis and light and dark respiration in freshwater algae. Can J Bot. 1977;55:809–18. doi: 10.1139/b77-095

[50] Werner U, Bird P, Wild C, Ferdelman T, Polerecky L, Eickert G. Spatial patterns of aerobic and anaerobic mineralization rates and oxygen penetration dynamics in coral reef sediments. Mar Ecol Prog Ser. 2006;309:93–105. doi: 10.3354/meps309093

[51] Sørensen K, Glazer B, Hannides A, Gaidos E. Spatial structure of the microbial community in sandy carbonate sediment. Mar Ecol Prog Ser. 2007;346:61–74. doi: 10.3354/meps06996

[52] Blott SJ, Pye K. GRADISTAT: A grain size distribution and statistics package for the analysis of unconsolidated sediments. Earth Surf Processes Landf. 2001;26(11):1237–48. doi: 10.1002/esp.261

[53] Folk R, Ward W. Brazos river bar: A study in the significance of grain size parameter. J Sediment Petrol. 1957;27:3–26.

[54] Krumbein W. Size frequency distributions of sediments and the normal phi curve. J Sediment Res. 1938;8:84–90.

[55] Davis BE. Loss-on-Ignition as an Estimate of Soil Organic Matter. Soil Sci Soc Am J. 1974;38:150–1. doi: 10.2136/sssaj1974.03615995003800010046x

[56] Hoogsteen MJJ, Lantinga EA, Bakker EJ, Tittonell PA. An evaluation of the loss-on-ignition method for determining the soil organic matter content of calcareous soils. Communications in Soil Science and Plant Analysis. 2018;49(13):1541–52. doi: 10.1080/00103624.2018.1474475

[57] Klindworth A, Pruesse E, Schweer T, Peplies J, Quast C, Horn M, et al. Evaluation of general 16S ribosomal RNA gene PCR primers for classical and next-generation sequencing-based diversity studies. Nucleic Acids Res. 2013;41(1):e1. doi: 10.1093/nar/gks808 22933715 PMC3592464

[58] Fadeev E, Cardozo-Mino MG, Rapp JZ, Bienhold C, Salter I, Salman-Carvalho V, et al. Comparison of Two 16S rRNA Primers (V3-V4 and V4-V5) for Studies of Arctic Microbial Communities. Front Microbiol. 2021;12:637526. doi: 10.3389/fmicb.2021.637526 33664723 PMC7920977

[59] Martin M. Cutadapt removes adapter sequences from high-throughput sequencing reads. EMBnet.j. 2011.

[60] Python Software Foundation. 2021. https://www.python.org/downloads/release/python-392/

[61] Callahan BJ, McMurdie PJ, Rosen MJ, Han AW, Johnson AJA, Holmes SP. DADA2: High-resolution sample inference from Illumina amplicon data. Nat Methods. 2016;13(7):581–3. doi: 10.1038/nmeth.3869 27214047 PMC4927377

[62] Gao X, Lin H, Revanna K, Dong Q. A Bayesian taxonomic classification method for 16S rRNA gene sequences with improved species-level accuracy. BMC Bioinformatics. 2017;18(1):247. doi: 10.1186/s12859-017-1670-4 28486927 PMC5424349

[63] Parks DH, Chuvochina M, Rinke C, Mussig AJ, Chaumeil P-A, Hugenholtz P. GTDB: an ongoing census of bacterial and archaeal diversity through a phylogenetically consistent, rank normalized and complete genome-based taxonomy. Nucleic Acids Res. 2021;50: D785–94. doi: 10.1093/nar/gkab776 PMC8728215 34520557

[64] Katoh K, Standley DM. MAFFT multiple sequence alignment software version 7: Improvements in performance and usability. Mol Biol Evol. 2013;30(4):772–80. doi: 10.1093/molbev/mst010 23329690 PMC3603318

[65] Price MN, Dehal PS, Arkin AP. FastTree 2--approximately maximum-likelihood trees for large alignments. PLoS One. 2010;5(3):e9490. doi: 10.1371/journal.pone.0009490 20224823 PMC2835736

[66] Miller MA, Pfeiffer W, Schwartz T. Creating the CIPRES Science Gateway for inference of large phylogenetic trees. 2010 Gateway Computing Environments Workshop (GCE), 2010. 1–8. doi: 10.1109/gce.2010.5676129

[67] Davis NM, Proctor DM, Holmes SP, Relman DA, Callahan BJ. Simple statistical identification and removal of contaminant sequences in marker-gene and metagenomics data. Microbiome. 2018;6(1):226. doi: 10.1186/s40168-018-0605-2 30558668 PMC6298009

[68] Oksanen J, Simpson G, Blanchet F, Kindt R, Legendre P, Minchin P. vegan: Community Ecology Package. 2025.

[69] Liu C, Cui Y, Li X, Yao M. microeco: An R package for data mining in microbial community ecology. FEMS Microbiol Ecol. 2021;97(2):fiaa255. doi: 10.1093/femsec/fiaa255 33332530

[70] Lozupone C, Lladser ME, Knights D, Stombaugh J, Knight R. ISME J. 2011;5: 169–72. doi: 10.1038/ismej.2010.133 20827291 PMC3105689

[71] Yan L. ggvenn: Draw Venn Diagram by “ggplot2.”. https://CRAN.R-project.org/package=ggvenn. 2023.

[72] Wei T, Simko V. R package “corrplot”: Visualization of a Correlation Matrix. https://github.com/taiyun/corrplot

[73] Wickham H. ggplot2. Cham: Springer International Publishing. 2016. doi: 10.1007/978-3-319-24277-4

[74] Love MI, Huber W, Anders S. Moderated estimation of fold change and dispersion for RNA-seq data with DESeq2. Genome Biol. 2014;15(12):550. doi: 10.1186/s13059-014-0550-8 25516281 PMC4302049

[75] Zhu A, Ibrahim JG, Love MI. Heavy-tailed prior distributions for sequence count data: Removing the noise and preserving large differences. Bioinformatics. 2019;35(12):2084–92. doi: 10.1093/bioinformatics/bty895 30395178 PMC6581436

[76] Webster N, Gorsuch H. Monitoring additional values within the Reef 2050 Integrated Monitoring and Reporting Program. Townsville: Great Barrier Reef Marine Park Authority, Australian Government. 2019.

[77] Probandt D, Eickhorst T, Ellrott A, Amann R, Knittel K. Microbial life on a sand grain: From bulk sediment to single grains. ISME J. 2018;12(2):623–33. doi: 10.1038/ismej.2017.197 29192905 PMC5776476

[78] Ahmerkamp S, Marchant HK, Peng C, Probandt D, Littmann S, Kuypers MMM, et al. The effect of sediment grain properties and porewater flow on microbial abundance and respiration in permeable sediments. Sci Rep. 2020;10(1):3573. doi: 10.1038/s41598-020-60557-7 32107429 PMC7046789

[79] Tout J, Jeffries TC, Webster NS, Stocker R, Ralph PJ, Seymour JR. Variability in microbial community composition and function between different niches within a coral reef. Microbial Ecology. 2014;67:540–52. doi: 10.1007/s00248-013-0362-5 24477921

[80] Littman RA, Willis BL, Pfeffer C, Bourne DG. Diversities of coral-associated bacteria differ with location, but not species, for three acroporid corals on the Great Barrier Reef. FEMS Microbiol Ecol. 2009;68(2):152–63. doi: 10.1111/j.1574-6941.2009.00666.x 19302548

[81] Rohwer F, Seguritan V, Azam F, Knowlton N. Diversity and distribution of coral-associated bacteria. Mar Ecol Prog Ser. 2002;243:1–10. doi: 10.3354/meps243001

[82] Ziegler M, Grupstra CGB, Barreto MM, Eaton M, BaOmar J, Zubier K, et al. Coral bacterial community structure responds to environmental change in a host-specific manner. Nat Commun. 2019;10(1):3092. doi: 10.1038/s41467-019-10969-5 31300639 PMC6626051

[83] Zhao W, Chen L, Huang X, Liu J, Niu W, Zhang XH. Distinct diversity, assembly, and co-occurrence patterns of the prokaryotic microbiome in coral ecosystems of the South China Sea. Ecol Indic. 2024;166:112452. doi: 10.1016/j.ecolind.2024.112452

[84] Webster NS, Taylor MW. Marine sponges and their microbial symbionts: Love and other relationships. Environ Microbiol. 2012;14(2):335–46. doi: 10.1111/j.1462-2920.2011.02460.x 21443739

[85] Pohlner M, Dlugosch L, Wemheuer B, Mills H, Engelen B, Reese BK. The majority of active rhodobacteraceae in marine sediments belong to uncultured genera: A molecular approach to link their distribution to environmental conditions. Front Microbiol. 2019;10:659. doi: 10.3389/fmicb.2019.00659 31001232 PMC6454203

[86] Simon M, Scheuner C, Meier-Kolthoff JP, Brinkhoff T, Wagner-Döbler I, Ulbrich M, et al. Phylogenomics of Rhodobacteraceae reveals evolutionary adaptation to marine and non-marine habitats. ISME J. 2017;11(6):1483–99. doi: 10.1038/ismej.2016.198 28106881 PMC5437341

[87] Williams TJ, Joux F, Lauro FM, Matallana-Surget S, Cavicchioli R. Physiology of Marine Oligotrophic Ultramicrobacteria. Extremophiles Handbook. Springer Japan. 2011. 1179–99. doi: 10.1007/978-4-431-53898-1_57

[88] Newton RJ, Griffin LE, Bowles KM, Meile C, Gifford S, Givens CE, et al. Genome characteristics of a generalist marine bacterial lineage. ISME J. 2010;4(6):784–98. doi: 10.1038/ismej.2009.150 20072162

[89] Durham BP, Grote J, Whittaker KA, Bender SJ, Luo H, Grim SL, et al. Draft genome sequence of marine alphaproteobacterial strain HIMB11, the first cultivated representative of a unique lineage within the Roseobacter clade possessing an unusually small genome. Stand Genomic Sci. 2014;9(3):632–45. doi: 10.4056/sigs.4998989 25197450 PMC4148974

[90] Sun F, Wang Y, Jiang Z, Sun C, Wang Y, Wu M. Abundance and diversity of dimethylsulfoniopropionate degradation genes of roseobacter group in the northern South China Sea. Frontiers in Marine Science. 2022;9. doi: 10.3389/fmars.2022.895613

[91] Cunliffe M. Correlating carbon monoxide oxidation with cox genes in the abundant Marine Roseobacter Clade. ISME J. 2011;5(4):685–91. doi: 10.1038/ismej.2010.170 21068776 PMC3105738

[92] Moran MA, Belas R, Schell MA, González JM, Sun F, Sun S, et al. Ecological Genomics of Marine Roseobacters. Appl Environ Microbiol. 2007;73: 4559–4569. doi: 10.1128/AEM.02580-06 17526795 PMC1932822

[93] Wang H, Liu J, Guo Y, Chen Y, Zhang C, He S, et al. Taxonomic, genomic, and ecological insights into a novel Flavobacteriaceae strain from coastal tidal flats. BMC Microbiol. 2025;25(1):344. doi: 10.1186/s12866-025-04069-2 40442592 PMC12124053

[94] Wang YW, Zhang J, Wang SX, Du ZJ, Mu DS. Aequorivita vitellina sp. nov. and Aequorivita xiaoshiensis sp. nov., isolated from marine sediment. Int J Syst Evol Microbiol. 2023;73:005801. doi: 10.1099/ijsem.0.005801 36961878

[95] Chianese G, Esposito FP, Parrot D, Ingham C, de Pascale D, Tasdemir D. Linear aminolipids with moderate antimicrobial activity from the antarctic gram-negative bacterium aequorivita sp. Mar Drugs. 2018;16(6):187. doi: 10.3390/md16060187 29843452 PMC6025266

[96] Shade A, Handelsman J. Beyond the Venn diagram: The hunt for a core microbiome. Environ Microbiol. 2012;14(1):4–12. doi: 10.1111/j.1462-2920.2011.02585.x 22004523

[97] Leite DCA, Salles JF, Calderon EN, Castro CB, Bianchini A, Marques JA, et al. Coral bacterial-core abundance and network complexity as proxies for anthropogenic pollution. Front Microbiol. 2018;9:833. doi: 10.3389/fmicb.2018.00833 29755445 PMC5934943

[98] Pootakham W, Mhuantong W, Yoocha T, Putchim L, Jomchai N, Sonthirod C, et al. Heat-induced shift in coral microbiome reveals several members of the Rhodobacteraceae family as indicator species for thermal stress in Porites lutea. Microbiologyopen. 2019;8(12):e935. doi: 10.1002/mbo3.935 31544365 PMC6925168

[99] Pootakham W, Mhuantong W, Yoocha T, Putchim L, Sonthirod C, Naktang C, et al. High resolution profiling of coral-associated bacterial communities using full-length 16S rRNA sequence data from PacBio SMRT sequencing system. Sci Rep. 2017;7(1):2774. doi: 10.1038/s41598-017-03139-4 28584301 PMC5459821

[100] Neave MJ, Rachmawati R, Xun L, Michell CT, Bourne DG, Apprill A, et al. Differential specificity between closely related corals and abundant Endozoicomonas endosymbionts across global scales. ISME J. 2017;11(1):186–200. doi: 10.1038/ismej.2016.95 27392086 PMC5335547

[101] Dubé CE, Ziegler M, Mercière A, Boissin E, Planes S, Bourmaud CA-F, et al. Naturally occurring fire coral clones demonstrate a genetic and environmental basis of microbiome composition. Nat Commun. 2021;12(1):6402. doi: 10.1038/s41467-021-26543-x 34737272 PMC8568919

[102] Brown BE, Bythell JC. Perspectives on mucus secretion in reef corals. Mar Ecol Prog Ser. 2005;296:291–309. doi: 10.3354/meps296291

[103] Wang J, Shen J, Wu Y, Tu C, Soininen J, Stegen JC, et al. Phylogenetic beta diversity in bacterial assemblages across ecosystems: Deterministic versus stochastic processes. ISME J. 2013;7(7):1310–21. doi: 10.1038/ismej.2013.30 23446837 PMC3695296

[104] Xu YF, Dong XM, Luo C, Ma SN, Xu JL, Cui YD. Nitrogen enrichment reduces the diversity of bacteria and alters their nutrient strategies in intertidal zones. Front Mar Sci. 2022. doi: 10.3389/fmars.2022.942074

[105] Chen J, McIlroy SE, Archana A, Baker DM, Panagiotou G. A pollution gradient contributes to the taxonomic, functional, and resistome diversity of microbial communities in marine sediments. Microbiome. 2019;7(1):104. doi: 10.1186/s40168-019-0714-6 31307536 PMC6632204

[106] Lian K, Liu F, Li Y, Wang C, Zhang C, McMinn A, et al. Environmental gradients shape microbiome assembly and stability in the East China sea. Environ Res. 2023;238(Pt 2):117197. doi: 10.1016/j.envres.2023.117197 37783325

[107] Donald L, Pipite A, Subramani R, Owen J, Keyzers RA, Taufa T. Streptomyces: Still the Biggest Producer of New Natural Secondary Metabolites, a Current Perspective. Microbiol Res. 2022;13:418–65. doi: 10.3390/microbiolres13030031

[108] Qin Q-L, Li Y, Zhang Y-J, Zhou Z-M, Zhang W-X, Chen X-L, et al. Comparative genomics reveals a deep-sea sediment-adapted life style of Pseudoalteromonas sp. SM9913. ISME J. 2011;5(2):274–84. doi: 10.1038/ismej.2010.103 20703316 PMC3105692

[109] Wang FY, Liu MY. Microbial community diversity of coral reef sediments on Liuqiu Island, southwestern Taiwan. J Mar Sci Eng. 2023;11:85. doi: 10.3390/jmse11010085

[110] Hemkemeyer M, Dohrmann AB, Christensen BT, Tebbe CC. Bacterial Preferences for Specific Soil Particle Size Fractions Revealed by Community Analyses. Front Microbiol. 2018;9:149. doi: 10.3389/fmicb.2018.00149 29527192 PMC5829042

[111] Lin J, Zhou X, Lu X, Xu Y, Wei Z, Ruan A. Grain size distribution drives microbial communities vertically assemble in nascent lake sediments. Environ Res. 2023;227:115828. doi: 10.1016/j.envres.2023.115828 37011792

[112] Zhang X, Liu S, Jiang Z, Wu Y, Huang X. Gradient of microbial communities around seagrass roots was mediated by sediment grain size. Ecosphere. 2022;13(2). doi: 10.1002/ecs2.3942

