# Supplementary material for: Benthic bacteria communities of coral reefs are shaped by sediment properties rather than coral trophic state
Source: PLoS One. 2026 Apr 3;21(4):e0346135. doi: 10.1371/journal.pone.0346135 (PMC13048377; doi:10.1371/journal.pone.0346135)
Supplement: S1 Table — Showing the number of samples (n), minimum (min), maximum (max), mean and standard error (SE). (PDF) [file pone.0346135.s001.pdf]

S1 Table. Alpha diversity metrics for the *Porites lutea* coral-associated and sediment only control samples, including; the observed number of ASV's, chao1 richness, shannon diversity, and simpson diversity. Showing the number of samples (n), minimum (min), maximum (max), mean and standard error (SE).

Diversity Index	Sample type	n	Min	Max	Mean	SE
Observed ASVs	Coral	60	114.00	733.00	547.93	16.97
	Sediment	81	35.00	546.00	396.31	10.22
Chao1	Coral	60	119.50	1958.47	1048.96	44.81
	Sediment	81	35.00	945.49	580.90	18.15
Shannon	Coral	60	3.28	6.38	5.85	0.09
	Sediment	81	2.69	6.04	5.48	0.07
Simpson	Coral	60	0.75	1.00	0.987	0.005
	Sediment	81	0.72	1.00	0.985	0.004
