# Supplementary material for: Benthic bacteria communities of coral reefs are shaped by sediment properties rather than coral trophic state
Source: PLoS One. 2026 Apr 3;21(4):e0346135. doi: 10.1371/journal.pone.0346135 (PMC13048377; doi:10.1371/journal.pone.0346135)
Supplement: S2 Table — Missing data for one chamber at site 03 (03_8). (PDF) [file pone.0346135.s002.pdf]

**S2 Table.** Summary table for the environmental variables for the *Porites lutea* coral-associated samples. Missing data for one chamber at site 03 (03_8).

| Site_ Chamber ID | Coral surface area ($m^2$) | Gross photosynthesis rates (g $O_2$ $m^{-2}$ $h^{-1}$) | Respiration rates (g $O_2$ $m^{-2}$ $h^{-1}$) | Net rate (g $O_2$ $m^{-2}$ $h^{-1}$) | P:R ratio | Total carbon (%) | Total nitrogen (%) | Total organic carbon (%) | C:N ratio | Mean grain size (φ) | Gravel content (g) | Gravel (%) | Sand (%) | Mud (%) |
|---|---|---|---|---|---|---|---|---|---|---|---|---|---|---|
| 1_7 | 0.07 | 0.50 | 0.20 | 0.30 | 2.54 | 10.61 | 0.04 | 0.08 | 1.85 | 1.41 | 9.43 | 0.01 | 0.96 | 0.04 |
| 1_8 | 0.08 | 0.54 | 0.17 | 0.37 | 3.13 | 10.42 | 0.05 | 0.09 | 1.90 | 2.47 | 10.95 | 0.00 | 0.84 | 0.16 |
| 3_7 | 0.03 | 0.38 | 0.12 | 0.25 | 3.05 | 9.64 | 0.06 | 0.09 | 1.60 | 0.95 | 36.01 | 0.01 | 0.95 | 0.03 |
| 3_9 | 0.02 | 0.36 | 0.14 | 0.22 | 2.56 | 9.94 | 0.06 | 0.09 | 1.68 | 1.45 | 29.14 | 0.00 | 0.93 | 0.06 |
| 5_7 | 0.02 | 0.40 | 0.10 | 0.29 | 3.88 | 11.42 | 0.04 | 0.09 | 2.04 | 2.44 | 11.28 | 0.00 | 0.81 | 0.19 |
| 6_7 | 0.03 | 0.49 | 0.25 | 0.23 | 1.94 | 11.34 | 0.03 | 0.09 | 2.60 | 1.31 | 18.00 | 0.00 | 0.98 | 0.02 |
| 6_8 | 0.08 | 0.11 | 0.01 | 0.10 | 8.66 | 11.34 | 0.04 | 0.29 | 6.41 | 1.62 | 37.80 | 0.00 | 0.97 | 0.03 |
| 6_9 | 0.11 | 0.56 | 0.28 | 0.28 | 2.03 | 11.35 | 0.04 | 0.09 | 2.26 | 1.27 | 21.81 | 0.01 | 0.95 | 0.04 |
| 10_7 | 0.05 | 0.28 | 0.11 | 0.17 | 2.63 | 11.16 | 0.05 | 0.09 | 1.69 | 1.50 | 11.15 | 0.02 | 0.95 | 0.03 |
| 10_8 | 0.07 | 0.33 | 0.13 | 0.21 | 2.65 | 8.59 | 0.04 | 0.10 | 2.26 | 1.51 | 6.13 | 0.01 | 0.96 | 0.04 |
| 13_9 | 0.38 | 0.73 | 0.28 | 0.45 | 2.59 | 11.10 | 0.03 | 0.08 | 2.57 | 1.04 | 9.96 | 0.00 | 0.98 | 0.01 |
| 15_7 | 0.06 | 0.59 | 0.19 | 0.41 | 3.20 | 10.54 | 0.04 | 0.09 | 2.30 | 1.54 | 18.26 | 0.01 | 0.95 | 0.04 |
| 15_8 | 0.10 | 0.52 | 0.24 | 0.28 | 2.14 | 11.27 | 0.04 | 0.09 | 2.25 | 1.82 | 3.56 | 0.00 | 0.95 | 0.05 |
| 15_9 | 0.34 | 0.78 | 0.38 | 0.40 | 2.04 | 10.12 | 0.04 | 0.09 | 2.37 | 1.68 | 7.08 | 0.00 | 0.98 | 0.02 |
| A1_8 | 0.02 | 0.35 | 0.17 | 0.18 | 2.08 | 11.40 | 0.04 | 0.09 | 2.53 | 1.45 | 27.83 | 0.01 | 0.97 | 0.02 |
| A1_9 | 0.02 | 0.49 | 0.20 | 0.30 | 2.51 | 11.45 | 0.04 | 0.10 | 2.21 | 0.53 | 25.97 | 0.08 | 0.88 | 0.04 |
| B6_7 | 0.07 | 0.34 | 0.14 | 0.20 | 2.44 | 11.17 | 0.03 | 0.09 | 3.25 | 1.12 | 7.86 | 0.00 | 0.99 | 0.01 |
| B6_8 | 0.62 | 1.29 | 0.53 | 0.76 | 2.44 | 11.27 | 0.04 | 0.09 | 2.19 | 1.62 | 16.62 | 0.00 | 0.97 | 0.03 |
| B6_9 | 0.05 | 0.27 | 0.11 | 0.15 | 2.35 | 10.97 | 0.03 | 0.09 | 3.34 | 1.30 | 5.28 | 0.00 | 0.98 | 0.02 |
