# Supplementary material for: Benthic bacteria communities of coral reefs are shaped by sediment properties rather than coral trophic state
Source: PLoS One. 2026 Apr 3;21(4):e0346135. doi: 10.1371/journal.pone.0346135 (PMC13048377; doi:10.1371/journal.pone.0346135)
Supplement: S3 Table — DESEq2 was run for each covariate in a single-term model, and log2 Fold Changes were shrunk using the apeglm method. The table shows the top three positive and negative significantly differentially abundant taxa with adjusted p values, merged with taxonomic annotations to genus, as well as the respective ASV. Positive log2 Fold Changes indicate higher abundance with increasing covariate values, while negative values indicate decreasing abundance. Continuous environmental covariates were Z-scaled prior to analysis. Scientific numbers used for log2 Fold Changes to show small numbers to 2 decimal places. (PDF) [file pone.0346135.s003.pdf]

S3 Table. Results of differential abundance of bacterial taxa across environmental covariates, identified using DESEq2 for the *Porites lutea* coral-associated samples. DESEq2 was run for each covariate in a single-term model, and log2 fold changes were shrunk using the apeglm method. The table shows the top three positive and negative significantly differentially abundant taxa with adjusted p values, merged with taxonomic annotations to family level. Positive log2 fold changes indicate higher abundance with increasing covariate values, while negative values indicate decreasing abundance. Continuous environmental covariates were Z-scaled prior to analysis. Scientific numbers used for log2 fold changes to show small numbers to 2 decimal places.

Covariate	Log2 Fold Change	padj	Phylum	Class	Order	Family
Gross photosynthesis	3.53	0.0001	Proteobacteria	Gammaproteobacteria	Woeseiales	Woeseiaceae
	1.74	0.0219	Planctomycetota	Planctomycetia	Pirellulales	Pirellulaceae
	2E-06	0.0000	Bacteroidota	Bacteroidia	Flavobacteriales	Flavobacteriaceae
	-1E-06	0.0000	Proteobacteria	Gammaproteobacteria	Chromatiales	Sedimenticolaceae
	-2E-06	0.0070	Actinobacteriota	Actinomycetia	Streptomycetales	Streptomycetaceae
	-3E-06	0.0002	Proteobacteria	Alphaproteobacteria	Rhodobacterales	Rhodobacteraceae
Respiration rate	2.84	0.0006	Planctomycetota	Planctomycetia	Pirellulales	Pirellulaceae
	1.82	0.0343	Proteobacteria	Gammaproteobacteria	Chromatiales	Chromatiaceae
	2E-06	0.0244	Proteobacteria	Gammaproteobacteria	Woeseiales	Woeseiaceae
	-2E-06	0.0193	Actinobacteriota	Actinomycetia	Streptomycetales	Streptomycetaceae
	-2E-06	0.0288	Proteobacteria	Gammaproteobacteria	Chromatiales	Sedimenticolaceae
	-1E-05	0.0287	Actinobacteriota	Acidimicrobiia	Acidimicrobiales	Ilumatobacteraceae
Total carbon	13.09	0.0000	Proteobacteria	Gammaproteobacteria	Chromatiales	Sedimenticolaceae
	9.61	0.0000	Proteobacteria	Alphaproteobacteria	Rhodospirillales	UXAT02
	2.43	0.0001	Bacteroidota	Bacteroidia	Flavobacteriales	Flavobacteriaceae
	-2E-06	0.0000	Planctomycetota	Phycisphaerae	Phycisphaerales	SM1A02
	-2.57	0.0235	Proteobacteria	Gammaproteobacteria	Woeseiales	Woeseiaceae
	-6.84	0.0000	Proteobacteria	Alphaproteobacteria	Rhodobacterales	Rhodobacteraceae
Total nitrogen	9.07	0.0000	Proteobacteria	Alphaproteobacteria	Rhodobacterales	Rhodobacteraceae
	8.59	0.0000	Bacteroidota	Bacteroidia	Flavobacteriales	Flavobacteriaceae
	6.68	0.0000	Acidobacteriota	Mor1	Mor1	Mor1
	-9.53	0.0000	Proteobacteria	Alphaproteobacteria	Rhodobacterales	Rhodobacteraceae
	-9.91	0.0000	Proteobacteria	Gammaproteobacteria	Enterobacterales	Enterobacteriaceae
	-11.32	0.0000	Proteobacteria	Gammaproteobacteria	Enterobacterales	Alteromonadaceae
C:N ratio	16.35	0.0000	Proteobacteria	Gammaproteobacteria	Enterobacterales	Alteromonadaceae
	11.70	0.0000	Bacteroidota	Bacteroidia	Flavobacteriales	Schleiferiaceae
	3E-06	0.0425	Bacteroidota	Bacteroidia	Bacteroidales	Marinifilaceae
	-2E-06	0.0086	Desulfobacterota	Desulfobulbia	Desulfobulbales	Desulfocapsaceae
	-1.48	0.0032	Proteobacteria	Alphaproteobacteria	Rhodobacterales	Rhodobacteraceae
	-7.42	0.0000	Actinobacteriota	Acidimicrobiia	Acidimicrobiales	Ilumatobacteraceae

Mean grain size	5.07	0.0000	Proteobacteria	Alphaproteobacteria	Rhizobiales	Rhizobiaceae
	4.86	0.0000	Patescibacteria	Paceibacteria	UBA9983_A	UBA918
	4.82	0.0000	Proteobacteria	Alphaproteobacteria	Rhodobacterales	Rhodobacteraceae
	-14.39	0.0000	Proteobacteria	Gammaproteobacteria	Enterobacterales	Enterobacteriaceae
	-17.55	0.0000	Proteobacteria	Gammaproteobacteria	Enterobacterales	Alteromonadaceae
	-19.96	0.0000	Bacteroidota	Bacteroidia	Flavobacteriales	Schleiferiaceae
Gravel (g)	7.33	0.0010	Proteobacteria	Alphaproteobacteria	Rhizobiales	Rhizobiaceae
	7.10	0.0059	Bacteroidota	Bacteroidia	Flavobacteriales	Flavobacteriaceae
	6.34	0.0000	Proteobacteria	Alphaproteobacteria	Rhodobacterales	Rhodobacteraceae
	-5.58	0.0007	Bacteroidota	Bacteroidia	Flavobacteriales	Schleiferiaceae
	-6.12	0.0000	Proteobacteria	Gammaproteobacteria	Enterobacterales	Enterobacteriaceae
	-8.58	0.0000	Proteobacteria	Gammaproteobacteria	Enterobacterales	Alteromonadaceae
Sand %	26.33	0.0000	Proteobacteria	Gammaproteobacteria	Enterobacterales	Alteromonadaceae
	24.19	0.0000	Proteobacteria	Alphaproteobacteria	UBA8366	GCA-2696645
	6.99	0.0000	Proteobacteria	Gammaproteobacteria	Pseudomonadales	Halieaceae
	-6.73	0.0491	Desulfobacterota	Desulfobacteria	Desulfobacterales	UBA11574
	-6.89	0.0417	Actinobacteriota	Acidimicrobiia	Acidimicrobiales	SKKL01
	-8.68	0.0000	Proteobacteria	Alphaproteobacteria	Rhodospirillales	UXAT02
Mud %	1.42	0.0112	Firmicutes	Bacilli	Bacillales_D	Amphibacillaceae
	0.77	0.0023	Actinobacteriota	Actinomycetia	Mycobacteriales	Nakamurellaceae
	2E-06	0.0133	Firmicutes	Bacilli	Bacillales_D	Amphibacillaceae
	-4.01	0.0000	Proteobacteria	Alphaproteobacteria	Rhodobacterales	Rhodobacteraceae
	-18.19	0.0000	Bacteroidota	Bacteroidia	Flavobacteriales	Schleiferiaceae
	-28.26	0.0000	Proteobacteria	Alphaproteobacteria	UBA8366	GCA-2696645
