# Supplementary figures and images for: Benthic bacteria communities of coral reefs are shaped by sediment properties rather than coral trophic state
Source: PLoS One. 2026 Apr 3;21(4):e0346135. doi: 10.1371/journal.pone.0346135 (PMC13048377; doi:10.1371/journal.pone.0346135)

(A)

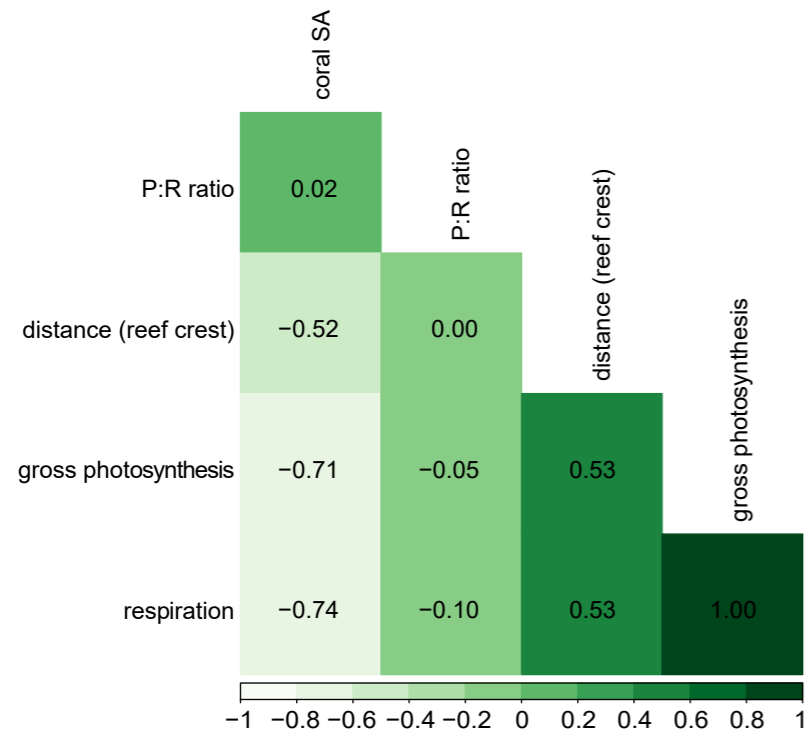

(B)

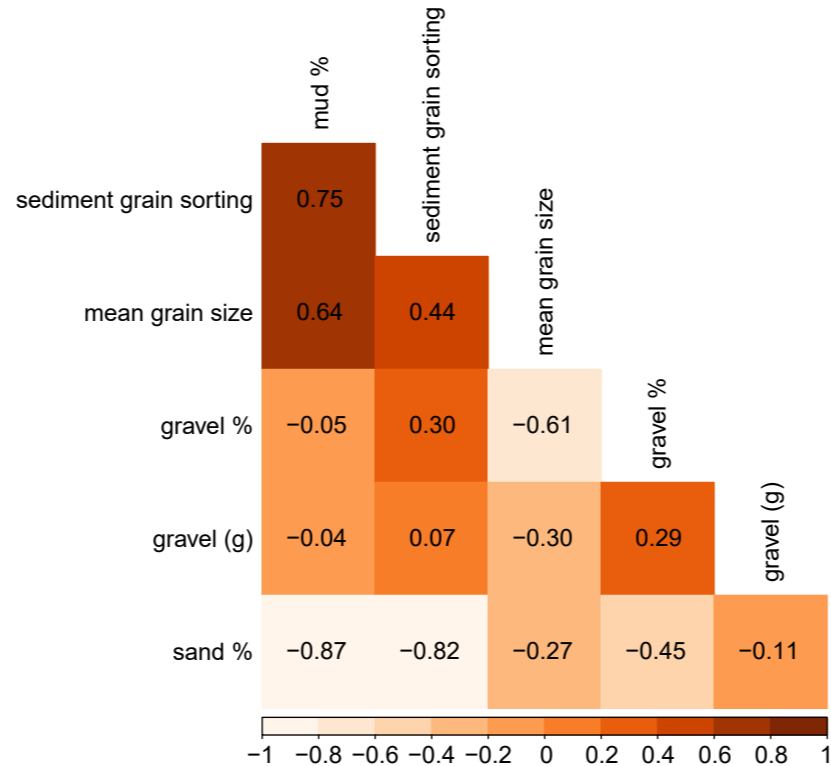

(C)

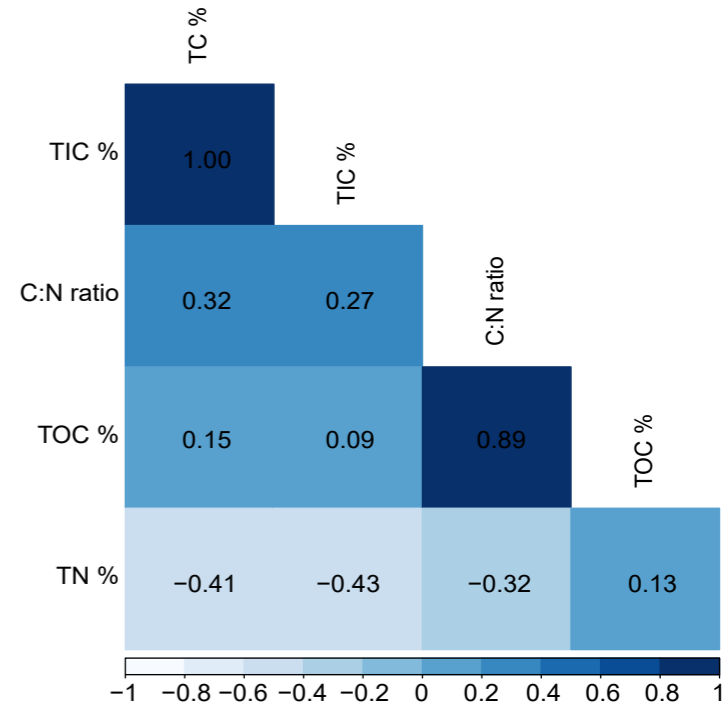

Supplement: S1 Fig — Colour intensity represents the strength and direction of correlation Correlated variables were removed for dbRDA analyses. (PDF) [file pone.0346135.s004.pdf]

(A)

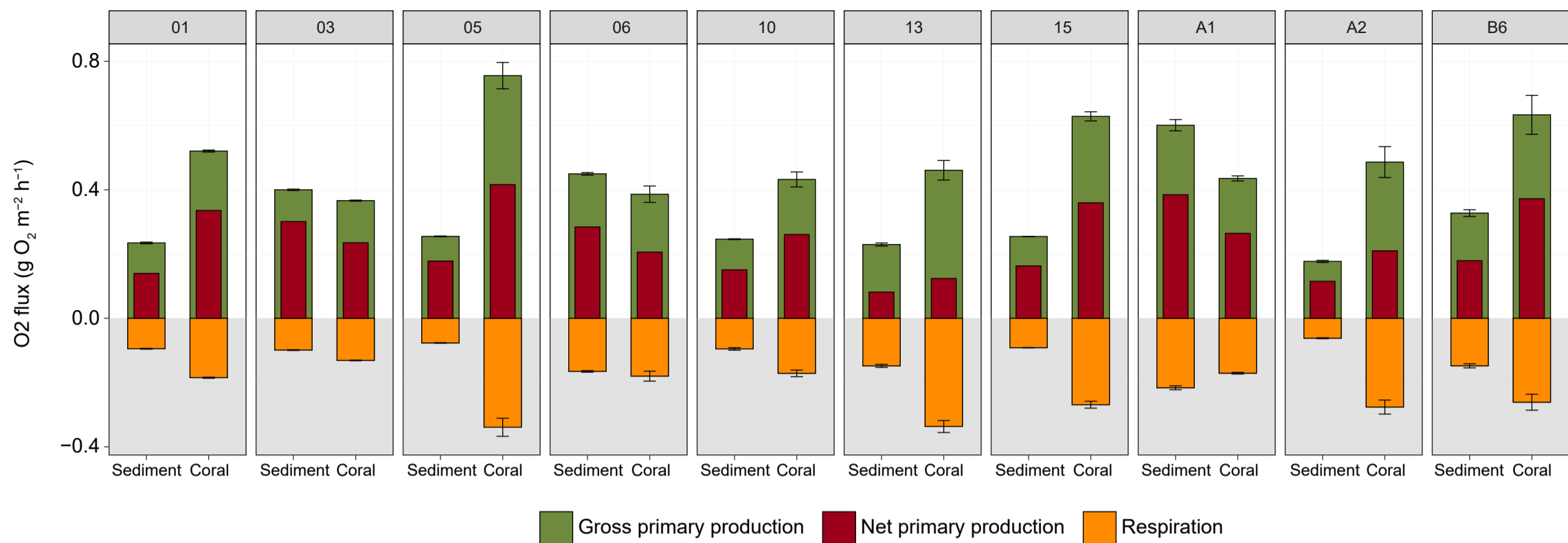

(B)

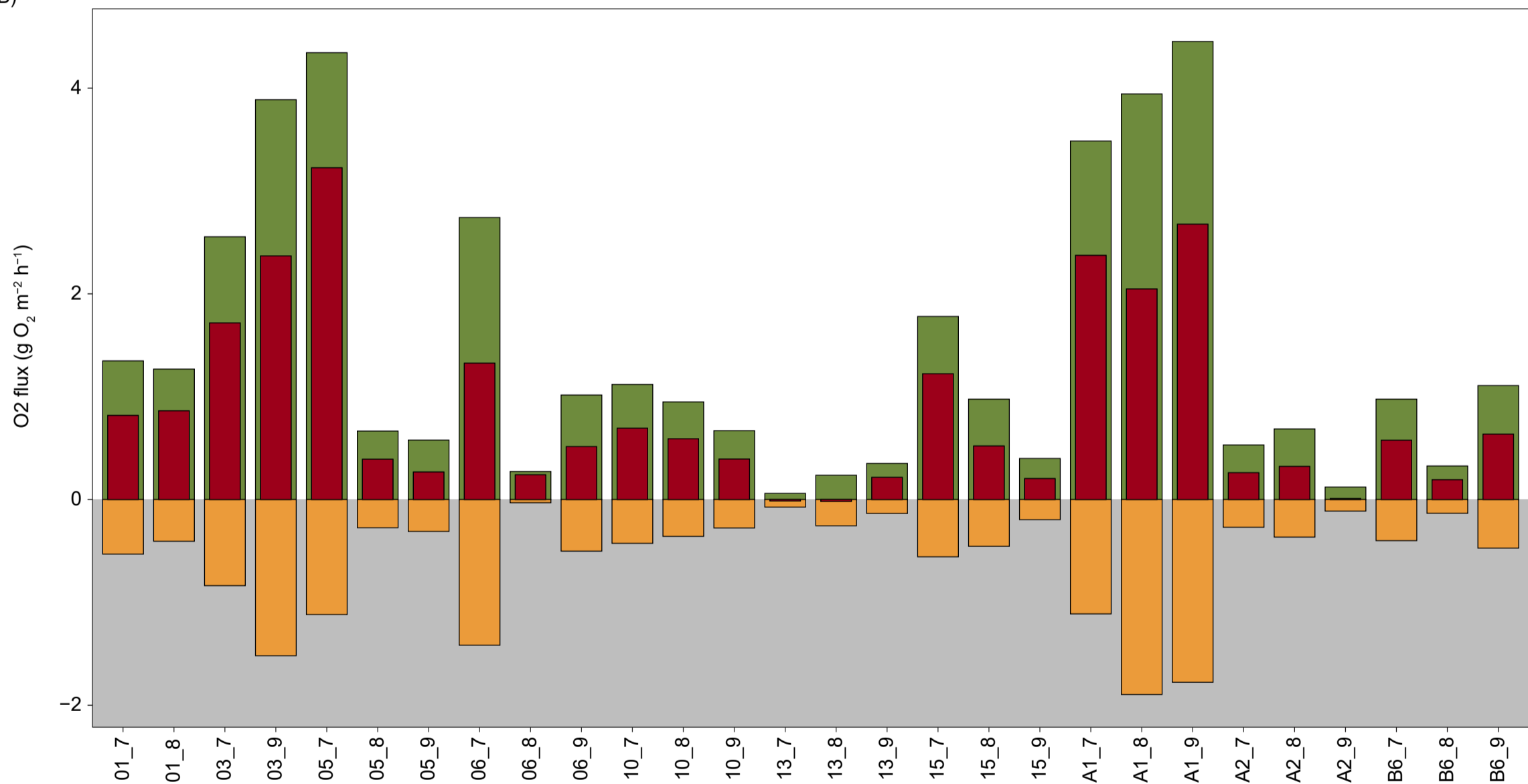

(C)

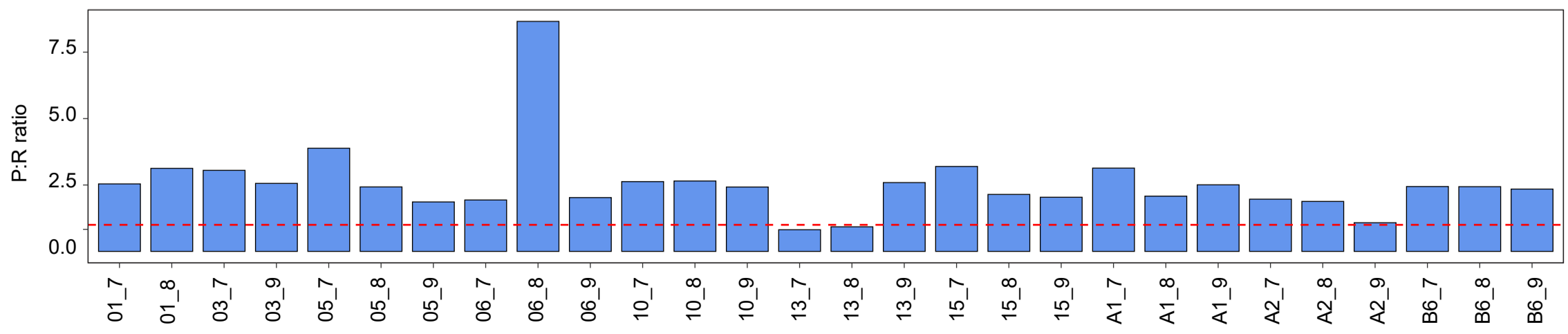

Supplement: S2 Fig — Both representing gross primary production rate (green bars), net primary production (red bars) and respiration rate (yellow bars). The net primary production was calculated by subtracting the respiration rate from the gross primary production rate. (C) The photosynthesis to respiration ratio was calculated for Porites lutea and used as a proxy for overall coral health. The red dashed line represents a P:R ratio of 1, where P:R > 1 indicates that autotrophic activity is higher than heterotrophic activity, meaning the system is a net producer of organic matter, and conversely when the P:R < 1 heterotrophic activity predominates over autotrophy. (PDF) [file pone.0346135.s005.pdf]

# Top responding bacterial families across environmental covariates

Family

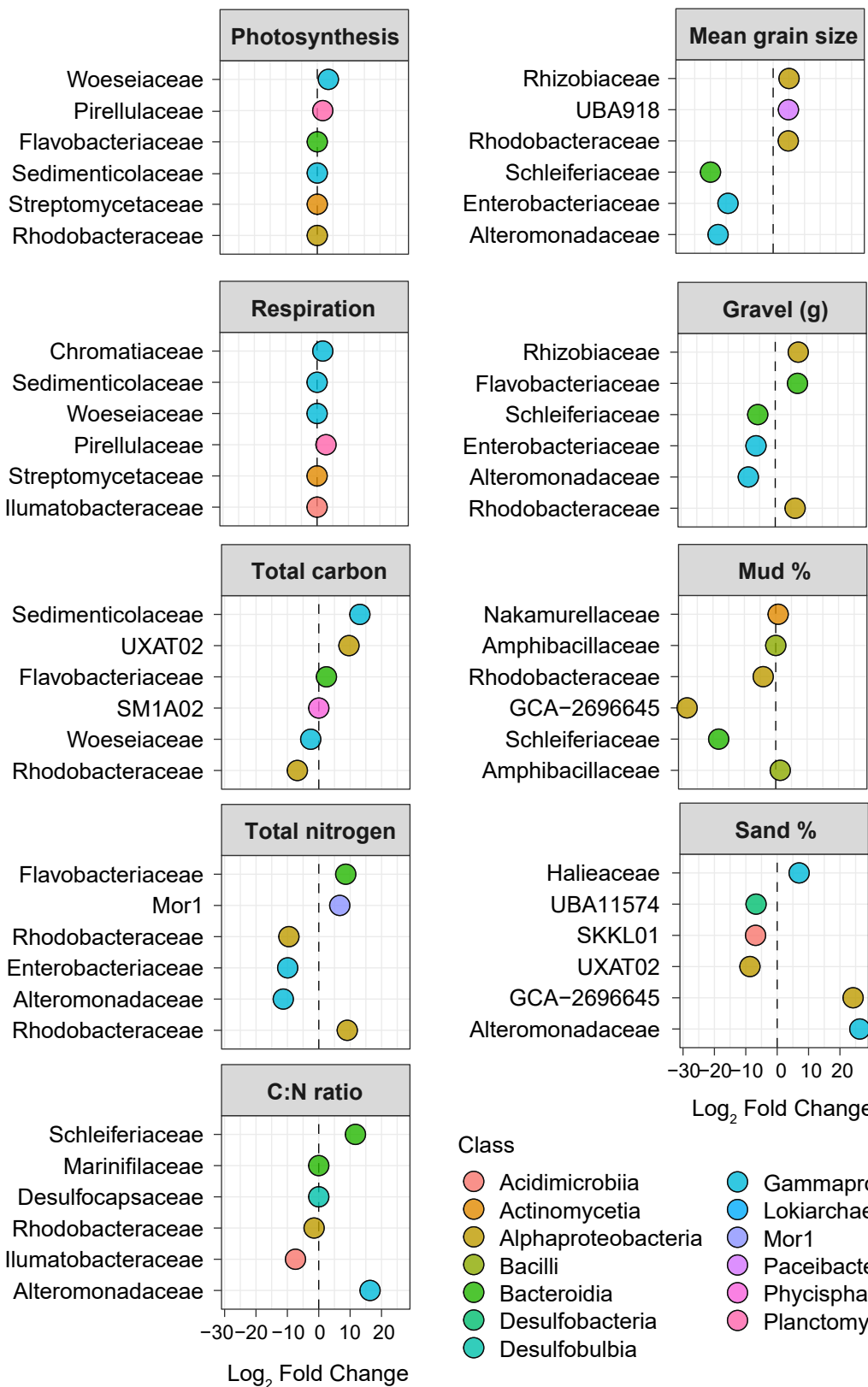

Supplement: S4 Fig — Points represent individual families (annotated with genus and ASV ID), coloured by bacterial class, with positive and negative log₂ fold changes indicating significantly higher or lower abundance with increasing covariate values, respectively. Horizontal dashed line at zero indicates no change in abundance. (PDF) [file pone.0346135.s007.pdf]
