# Supplementary material for: Benthic bacteria communities of coral reefs are shaped by sediment properties rather than coral trophic state
Source: PLoS One. 2026 Apr 3;21(4):e0346135. doi: 10.1371/journal.pone.0346135 (PMC13048377; doi:10.1371/journal.pone.0346135)
Supplement: S3 Fig — (A) Principal coordinates analysis (PCoA) using weighted UniFrac distances for the Porites lutea coral (triangle symbols) and sediment samples (circle symbols) for each site, with the ellipses around the sample type (i.e., coral-associated sediment or sediment control samples). (B) Top bacterial taxa (adjusted p ≤ 0.05) identified using DESeq2 differential abundance for the coral-associated sediment and sediment control samples. Points represent individual genera (annotated with family, genus and ASV ID), coloured by bacterial class, with positive log2 Fold Changes indicating these taxa were in higher abundance in the sediment control samples, while negative log₂ fold changes indicate taxa higher in abundance for the coral-associated sediment samples. Horizontal dashed line at zero indicates no change in abundance. (C) Core microbial community for the coral-associated sediment and sediment control samples, run at the taxonomic level of family. Insert (c) shows a Venn diagram for the coral-associated sediment and sediment controls with the numbers inside the sections representing the number of unique and shared families. (PDF) [file pone.0346135.s006.pdf]

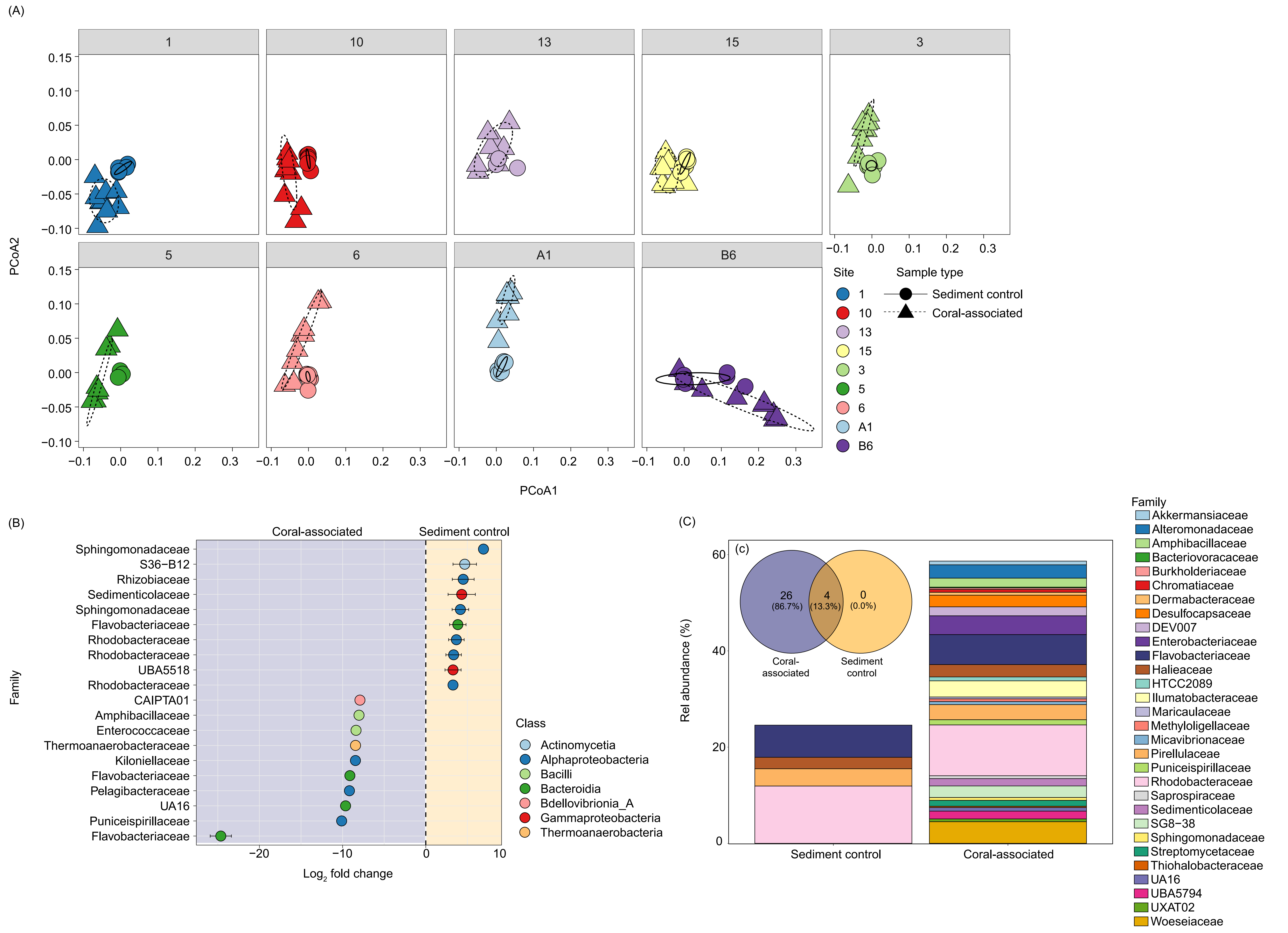
(A)
1
10
13
15
3
5
6
A1
B6
0.15
0.10
0.05
0.00
−0.05
−0.10
−0.1
0.0
0.1
0.2
0.3
PCoA2
PCoA1
Site
1
10
13
15
3
5
6
A1
B6
Sample type
Sediment control
Coral-associated
(B)
Coral-associated
Sediment control
Sphingomonadaceae
S36−B12
Rhizobiaceae
Sedimenticolaceae
Sphingomonadaceae
Flavobacteriaceae
Rhodobacteraceae
Rhodobacteraceae
UBA5518
Rhodobacteraceae
CAIPTA01
Amphibacillaceae
Enterococcaceae
Thermoanaerobacteraceae
Kiloniellaceae
Flavobacteriaceae
Pelagibacteraceae
UA16
Puniceispirillaceae
Flavobacteriaceae
Family
−20
−10
0
10
Log_2 fold change
Class
Actinomycetia
Alphaproteobacteria
Bacilli
Bacteroidia
Bdellovibrionia_A
Gammaproteobacteria
Thermoanaerobacteria
(C)
(c)
26
(86.7%)
4
(13.3%)
0
(0.0%)
Coral-
associated
Sediment
control
60
40
20
0
Rel abundance (%)
Sediment control
Coral-associated
Family
Akkermansiaceae
Alteromonadaceae
Amphibacillaceae
Bacteriovoracaceae
Burkholderiaceae
Chromatiaceae
Dermabacteraceae
Desulfocapsaceae
DEV007
Enterobacteriaceae
Flavobacteriaceae
Halieaceae
HTCC2089
Ilumatobacteraceae
Maricaulaceae
Methyloligellaceae
Micavibrionaceae
Pirellulaceae
Puniceispirillaceae
Rhodobacteraceae
Saprospiraceae
Sedimenticolaceae
SG8−38
Sphingomonadaceae
Streptomycetaceae
Thiohalobacteraceae
UA16
UBA5794
UXAT02
Woeseiaceae
